# A CD47 antibody with minimized erythrocyte and thrombocyte toxicities

**DOI:** 10.3389/fonc.2025.1686180

**Published:** 2025-11-26

**Authors:** Min Pei, Xiao-Dong Dai, Xiao-Fan Jiang, Cai-Yi Yuan, Jie Tan, Kai-Feng He, Guo-Jian Liu, Jin-Di Zhu, Huan-Huan Li, Ai-Ling Pan, Ya-Ru Wang, Yi-Li Chen, Xiao-Jia Chen, Chunhe Wang

**Affiliations:** 1 Institute of Biomedicine & Department of Cell Biology, College of Life Science and Technology, Jinan University, Guangzhou, China; 2 Department of Antibody Discovery, Shanghai Mabstone Biotechnology Ltd., Shanghai, China; 3 Biotherapeutics Discovery Research Center, Shanghai Institute of Materia Medica, Chinese Academy of Sciences, Shanghai, China; 4 School of Chinese Materia Medica, Nanjing University of Chinese Medicine, Nanjing, China; 5 Department of Research and Development Center, Dartsbio Pharmaceuticals Ltd., Zhongshan, Guangdong, China; 6 School of Life Science of Technology, ShanghaiTech University, Shanghai, China

**Keywords:** CD47, monoclonal antibody, macrophages, hematotoxicity, N-linked glycosylation

## Abstract

**Introduction:**

Blockade of the CD47/SIRPa axis has emerged as a promising approach to enhance macrophage-mediated anti-tumor activities in cancer immunotherapy. However, the clinical application of early CD47 antibodies has been associated with significant hematotoxicities, including hemagglutination, anemia, and thrombopenia. Although several CD47 antibodies have generally avoided hemagglutination, anemia, and thrombopenia remain, potentially mediated by enhanced phagocytosis of erythrocytes and thrombocytes.

**Methods:**

1B2–10 is a humanized CD47-neutralizing antibody generated by mouse immunization and phage display technology. Its efficacy and safety were evaluated using *in vitro* assays, tumor xenograft models, and studies in cynomolgus monkeys. *In silico* modeling and mutagenesis analyses were performed to identify the 1B2-10 binding epitopes.

**Results:**

*In vitro*, 1B2–10 exhibited minimized binding to erythrocytes and thrombocytes, did not induce hemagglutination, and reduced erythrocyte phagocytosis. It enhanced macrophage-mediated phagocytosis of tumor cells and showed potent antitumor effects as a monotherapy or in combination with a PD-1 antibody in tumor models. In cynomolgus monkeys, single doses of 1B2–10 at 120 mg/kg and repeated doses at 100 mg/kg were well-tolerated, with no significant hematological toxicities observed. *In silico* studies revealed the molecular basis for reduced erythrocyte binding, demonstrating that two critical N-linked glycans sterically masked epitopes.

**Discussion:**

These findings suggest that 1B2-10 demonstrates a favorable efficacy and safety profile, indicating great therapeutic potential for cancer. Based on these studies, 1B2–10 has been renamed as DS003 and is currently advancing through Phase I clinical trials in China to further evaluate its safety profile and preliminary efficacy in human subjects.

## Introduction

Cancer immunotherapy has revolutionized the oncology landscape by leveraging patients’ immune systems to combat tumor cells, but the high mortality rate associated with various cancers remains a significant challenge (1–3). Cancer cells evade host immune surveillance through induction of T cell tolerance as well as functional suppression of innate immune cells (4), underscoring the importance of stimulating both innate and adaptive immune mechanisms. Macrophages have emerged as a promising target for cancer immunotherapy, given their abundant presence within tumors, phagocytic capabilities, cytotoxin release, and antigen presentation functions that bridge the innate and adaptive immunities (5).

However, the abilities of macrophages to engage and eliminate malignant cells (6, 7) are undermined by the activation of the CD47- SIRPα (signal regulatory protein alpha) axis. CD47 is a transmembrane protein overexpressed on various tumor cells. By binding to SIRPα on the surface of myeloid cells, including macrophages, neutrophils, and dendritic cells, it transmits a “don’t eat me” signal, thereby inhibiting the innate immune-mediated phagocytosis of tumor cells (8) and enabling malignant cells to evade immune surveillance (9, 10). Multiple studies have demonstrated that blockade of CD47 enhances phagocytosis and antigen-presenting capabilities of macrophages and neutrophils, thus activating tumor-specific T cells and consequently stimulating adaptive immune responses (4, 11–13). This dual activation of both innate and adaptive immunities underscores the therapeutic potential of including the blockade of CD47 in comprehensive cancer management strategies.

Targeting CD47 as a cancer treatment strategy has been extensively investigated in clinical settings in the past few years. Various approaches to antagonize the CD47/SIRPα axis have been explored, including monoclonal antibodies (mAbs), bispecific antibodies (bsAbs), and fusion proteins. Despite promising results from these antagonists in preclinical studies, clinical development has been limited by the unsatisfactory efficacy, as well as by concerns of hematotoxicity, including anemia, thrombopenia, and neutropenia (14, 15) (**Supplementary Table 1**). The high expression level of CD47 on erythrocytes and thrombocytes renders these cells resistant to phagocytosis while blocking CD47 can break their protection and cause hematotoxicity (16, 17). Additionally, the binding of the bivalent antibodies to erythrocytes caused them to aggregate, called hemagglutination. CD47 antibodies, even in the human IgG4 subtype, can also harm blood cells by Fc-mediated antibody-dependent cellular cytotoxicity (ADCC) and complement-dependent cytotoxicity (CDC) functions.

The CD47 neutralizing mAb magrolimab (Hu5F9, or Hu5F9‐G4) was hemagglutination-prone and demonstrated significant hematologic toxicities in clinical trials. While the implementation of a priming and maintenance dosing strategy showed promise in reducing anemia incidence, the phase III ENHANCE trial (NCT04313881) for magrolimab was discontinued due to futility and severe adverse events (18–20). Subsequent CD47 mAbs generally avoided hemagglutination, but many of them still have erythrocyte binding (**Supplementary Table 1**). More importantly, none of them were tested for both erythrocyte phagocytosis and thrombocyte binding. Ligufalimab (AK117) is a CD47-neutralizing mAb that binds to human erythrocytes without inducing hemagglutination (21, 22). Unlike Hu5F9, AK117 does not require the administration of a lower “priming” dose to prevent anemia (23) and is tested in Phase II clinical trials in combination with various therapeutic agents. However, it induced phagocytosis of erythrocytes, while its binding to thrombocytes was not investigated. In cynomolgus monkeys, it caused the reduction of erythrocytes and thrombocytes, although at a level lower than Hu5F9. In the Phase I clinical trial, its maximum tolerable dose (MTD) was determined to be 20 mg/kg and the treatment-related adverse events included anemia, thrombopenia, lymphopenia, and neutropenia. AO-176 is another CD47-neutralizing antibody that does not bind to erythrocytes or induce hemagglutination *in vitro*, but binds to thrombocytes and was not tested for erythrocyte phagocytosis. Its clinical development came to a halt, presumably due to a still dissatisfying benefit/risk ratio (24–26). Similarly, the clinical development of lemzoparlimab (TJC4), which exhibited minimal erythrocyte binding and hemagglutination, but was not tested for erythrocyte phagocytosis or thrombocyte binding, was also suspended outside China (27–29). Therefore, the absence of hemagglutination may be necessary but not enough to significantly change the benefit/risk ratio of CD47 mAbs. In fact, hemagglutination, phagocytosis of erythrocytes and thrombocytes, ADCC, and CDC were all considered as contributing factors to hematologic toxicities, and assays for all of the above should be in place to screen for improved CD47-neutralizing mAbs.

In this study, we reported the discovery of 1B2-10, a CD47-neutralizing mAb, with no significant erythrocyte and thrombocyte binding, hemagglutination, erythrocyte phagocytosis, ADCC, or CDC. It enhanced macrophage-mediated phagocytosis of cancer cells *in vitro* and demonstrated potent antitumor efficacy as a monotherapy and in combination with a programmed death 1 (PD-1) antibody. In cynomolgus monkeys, 1B2–10 showed minimal impact on erythrocytes following single or repeated administrations and exhibited favorable pharmacokinetic and safety profiles. These findings laid the foundation for the clinical development of 1B2–10 as an efficacious and safer anti-tumor therapy.

## Materials and methods

### Cell lines

HEK293F cells were obtained from the ThermoFisher (#R79007), human tumor cell lines Daudi, HL-60, Raji, A549, SKBR-3, HT-29 and mouse monocytic macrophage leukemia cell line RAW264.7 were purchased from the cell bank of the Chinese Academy of Sciences (#TCHu140, #TCHu23, #TCHu44, #TCHu150, #TCHu225, #TCHu103, #TCM13). HEK293F cells were cultured in FreeStyle™ 293 medium (Gibco, #12338018). Daudi, HL-60 and Raji cells were cultured in RPMI1640 (Cytiva, #SH30027.02) with 10% fetal bovine serum (FBS) (Gibco, #A5256701). RAW264.7, A549, SKBR-3, and HT-29 cells were cultured in DMEM (Gibco, #10566016) with 10% FBS. All cells were cultured in a 37°C incubator with 5% CO_2_.

### Animals

Female, BALB/c nude mice and SCID/Beige mice (5–7 weeks old) were obtained from Beijing Vital River Laboratory Animal Technology Co., Ltd, BALB/c-hPD1/hSIRPα transgenic mice were purchased from GemPharmatech Co. Ltd. Animals were housed in specific pathogen-free conditions (20-26 °C, 40-70% humidity) with a 12-hour light cycle and ad libitum access to food and water. All animal care and experimental procedures adhered to the Guidelines for the Care and Use of Experimental Animals issued by the National Science and Technology Commission of the People’s Republic of China and approved by the Institutional Animal Care and Use Committee (IACUC) of Shanghai Model Organisms Center, Inc. (Approval No. 2021-0009-06).

### Proteins and antibodies

The human/cynomolgus CD47 or human SIRPα gene encoding the extracellular domain was fused to an Fc/his tag and subcloned into subcloned into pcDNA3.1(+) vector (Invitrogen, #V79020), followed by transiently transfecting HEK293F cells for fusion protein production. The anti‐CD47 antibody Hu5F9‐G4 was generated internally based on publicly available sequences. The recombinant proteins and antibodies were purified from the supernatant using Protein A columns (Bestchrom, #AA0273). The molecular weight and purity of the target proteins were assessed by SDS-PAGE. Fluorescent dye-labeled antibodies used for flow cytometry were purchased from BioLegend.

### Monoclonal antibody generation and humanization

Human CD47-targeting monoclonal antibodies were generated via a systematic approach. In brief, eight-week-old female Balb/c mice were immunized with purified CD47-hFc fusion protein emulsified in a 1:1 ratio of Freund’s complete and incomplete adjuvant (Sigma-Aldrich, #F5881, #F5506) through bi-weekly injections, totaling seven administrations. Splenocytes were harvested from mice demonstrating elevated serum titers, and mRNA was extracted. Subsequently, cDNA was synthesized by RT-PCR, and murine antibody heavy and light chain variable regions were amplified using mouse-specific universal primers for phage display library construction (30, 31). The resulting phage display library was then subjected to a single round of bio-panning. Human CD47-specific antibodies were subsequently identified through a combination of capture lift assay and single-point phage ELISA. Positive phage clones exhibiting both high binding affinity and thermostability were sequenced, yielding 14 unique CD47 antibody sequences. Following expression in HEK293F cells, the 14 purified murine antibodies were further screened using an *in vitro* human red blood cell (RBC) hemagglutination assay to isolate the 1B2 antibody, which did not induce hemagglutination. To humanize 1B2, a previously described protocol was implemented (32–34). Human antibody heavy and light chain sequences characterized by high usage frequency and favorable druggability profiles were selected as templates for amplifying framework regions. These human germline framework regions were then combined with the complementarity-determining regions (CDRs) from the murine antibody to construct a combinatorial phage library. Humanized antibodies were screened through affinity-based bio-panning and thermal stability assessment. The resultant humanized antibody was designated 1B2-10.

### Binding and blocking activity characterization by ELISA

Binding activity to CD47: 96-well high binding ELISA plates (Greiner Bio-One GmbH, Austria) were coated with antibody overnight at 4°C. After washed, stepwise dilutions of biotin-labeled human CD47-Fc or cynomolgus CD47-Fc were added and incubated for 1 hour. After another three times washing, wells were incubated with 1:2500 diluted Streptavidin-Horseradish peroxidase (HRP) (ThermoFisher, #N100) for 1 hour.

Blocking of SIRPα to CD47: 96 ELISA plate (Greiner, #650061) was coated with human SIRPα (ECD, Fc tag) overnight at 4°C. Serially diluted antibody was preincubated with biotin-labeled hCD47-Fc for 30 min and then the mixtures were added to wells to react for 1 hour. Then the wells were incubated with streptavidin-HRP (1:2500 dilution) for 30 min.

### Binding and blocking activity characterization by fluorescence-activated cell sorting

Binding assay: Tumor cells that expressed CD47 were harvested and incubated with serial dilutions of 1B2-10, Hu5F9-G4, or isotype-G4 at 4°C for 1 hour. After washing three times with wash buffer (1% FBS in PBS), the PE anti-human IgG Fc antibody (BioLegend, #366904) was adopted as a secondary antibody for fluorescence detection.

Blocking assay: CD47-expressing cell lines were harvested and incubated with biotin-labeled human SIRPα (ECD, Fc tag), followed by adding multiple dilutions of antibody 1B2-10, Hu5F9-G4, or isotype-G4. After incubating at 4°C for 1 hour, cells were rinsed thrice with wash buffer. PE-streptavidin (BioLegend, #405204) was added and incubated for 30 min. Finally, stained cells in binding and blocking assay were rewashed and suspended by PBS. Flow cytometry data were acquired using CytoFlex Flow Cytometer (Beckman Coulter, Inc.).

### Affinity determination by bio-layer interferometry

The affinity of 1B2–10 and Hu5F9-G4 to human CD47-his were measured using the Octet RED 96 platform (Fortebio). Antibodies were diluted to a concentration of 10 μg/ml and captured by protein A biosensors (Sartorius, #18-5010). Following equilibration in 0.05% PBST, antibody-loaded sensors were immersed in antigen solutions with concentrations ranging from 1.56 nM to 100 nM. Response curves were fitted using a 1:1 global fitting model in the manufacturer’s analysis software, and the kinetic constant (*KD*) was calculated after data processing.

### Erythrocytes and thrombocytes binding assay

Human whole blood samples were purchased from Schbio biotech (Shanghai) Co., LLC. Erythrocytes were isolated from the whole blood through centrifugation at 1000 rpm for 10 min, followed by three washes with PBS (Meilunbio, #PWL050). Human thrombocytes were isolated from thrombocyte-rich plasma (PRP) through a two-step centrifugation process: first, whole blood collected in citrate vacutainers was centrifuged at 1000 rpm to remove erythrocytes; subsequently, the PRP was centrifuged at 3000 rpm to obtain the thrombocyte pellet. The isolated thrombocytes were washed and pre-treated with human IgG4 isotype antibody to block Fc receptors.

Washed erythrocytes were incubated with gradient concentrations 1B2-10, Hu5F9-G4, or human IgG4 isotype at 4°C for 1 hour. After washing, the cells were incubated with PE-conjugated anti-human IgG Fc antibody at 4°C for 30 minutes. Samples were analyzed using a Beckman flow cytometer.

Isolated thrombocytes were incubated with gradient concentrations 1B2-10, Hu5F9-G4, or human IgG4 isotype at 37°C for 1 hour, washed and incubated with PE-conjugated anti-human IgG Fc antibody at 37°C for 30 minutes, and further stained with APC-conjugated anti-human CD41 and FITC-conjugated anti-human CD61 (BioLegend, #303709, #336403). Samples were analyzed using a Beckman flow cytometer gating on CD41^+^ CD61^+^ cells.

### Hemagglutination assay

Washed erythrocytes were diluted to a 2% suspension. Aliquots of 10 μL erythrocyte suspension were pipetted into a round-bottom 96-well cell culture plate (Corning, NY, USA). 1B2–10 and control antibodies were serially diluted to concentrations ranging from 13 nM to 3.3 μM. Subsequently, 20 μL of diluted antibody was added to each erythrocyte suspension, and the plate was incubated at 37°C for 2 hours. The plate was then scanned using an Epson Perfection V370 Photo Scanner (Epson (China) Co., Ltd.). Dispersed erythrocytes indicated hemagglutination, whereas a compact pellet at the bottom of the well represented the absence of hemagglutination.

### Phagocytosis assay

The murine macrophage cell line RAW264.7 was labeled with Cell Proliferation Dye eFluor™ 670 (Invitrogen, #65-0840-85). The stained macrophages were seeded into a 96-well ultra-low attachment plate (Corning, #7007). Concurrently, target cells were stained with carboxyfluorescein succinimidyl ester (CFSE) (Invitrogen, #C34554) and pre-incubated with 1B2–10 or control antibodies for 30 minutes. The mixtures of antibody and tumor cells were placed in a primed macrophage layer and co-cultured at a ratio of 4:1 for an additional 2 hours at 37°C, 5% CO_2_ incubator. For erythrocyte phagocytosis the target: effector ratio was 1:6. Particles exhibiting dual fluorescence signals were identified as tumor cells engulfed by macrophages. The phagocytosis index was calculated by dividing the percentage of double-positive cells by the total percentage of stained macrophages and multiplying the number by 100%.

### ADCC assay and CDC assay

The ADCC assay utilized the engineered NK92/CD16a stable cell line as effector cells and CD47-expressing HL-60 cells as target cells. Additionally, ch14.18 (dinutuximab, targeting GD2) served as a positive control antibody, with IMR32 cells as the corresponding target. The antibody concentrations were tested at three points, starting from 100 nM with tenfold serial dilutions. The effector-to-target cell ratio was maintained at 5:1 (E: T = 5:1). The cytotoxic effect on target cells was quantified using a lactate dehydrogenase assay (LDH-Glo™ Cytotoxicity Assay, Promega, #J2381).

For the CDC assay, complement was derived from normal human serum (NHS) at a final concentration of 8%. Parallel to the ADCC assay, antibody concentrations were evaluated at three points, beginning at 100 nM with tenfold serial dilutions. Target cell viability was assessed using a SpectraMax M5e Multi-Mode Microplate Reader. All data were subsequently analyzed using GraphPad Prism 8.0 ^®^ software.

### Tumor xenograft mouse model

Human leukemia cell xenograft models were established using Daudi cells in BALB/c nude mice and HL-60 cells in SCID mice via subcutaneous inoculation. Tumor cells (5×10^6^ cells) were suspended in PBS mixed with an equal volume of Matrigel (Corning, #356234), and then injected subcutaneously into the right flank of each mouse. When tumor volumes reached 100–200 mm^3^, animals were randomly divided into groups (10 animals/group). 1B2–10 was administered intraperitoneally at doses of 0.2, 1, or 5 mg/kg, or vehicle control, thrice weekly for three weeks. Tumor dimensions were measured using a vernier caliper before each dose administration. Tumor volume (mm^3^) was calculated using the formula: (length × width^2^ × 0.5). Relative tumor growth inhibition (TGI%) was calculated for each mouse individually. Animals were euthanized via controlled CO_2_ inhalation (20% flow rate of chamber volume per minute) when tumor size exceeded 2000 mm^3^, in compliance with ethical guidelines for animal welfare.

To evaluate the *in vivo* bioactivity of 1B2–10 in combination with an anti-PD-1 antibody, a mouse colon cancer model was established using CT26-hPDL1/hCD47 cells in BALB/c-hPD1/hSIRPα transgenic mice. Tumor cells were resuspended in PBS at 2×10^7^ cells/mL. Subsequently, 2×10^6^ cells were subcutaneously inoculated into each mouse. When tumor volumes reached 100–120 mm^3^, animals were randomly divided into groups (10 animals/group). 1B2–10 was administered in combination with an in-house anti-PD-1 antibody (17-D5) that does not cross-react to mouse PD-1, with human IgG4 isotype serving as a control. Tumor volumes and animal body weights were measured thrice weekly, while clinical symptoms were observed and documented daily.

### Pharmacokinetics and toxicity studies in cynomolgus monkeys

All cynomolgus monkey-related experiments were conducted at CTI Laboratories (Suzhou) following facility standard operating procedures. The experimental protocols were approved by the IACUC (Approval No. IACUC-A2021007-T014-01) and complied with all applicable animal welfare guidelines and ethical regulations.

For the pharmacokinetic study, a total of 18 cynomolgus monkeys randomly divided into 3 groups (3 animals/sex/group) were enrolled for this study and administered with 1B2–10 at 1, 5, and 20 mg/kg with a single intravenous infusion. The blood sample for concentration analyses was collected at pre-dose, and 15 min, 2h, 4h, 8h, 24 h, 48 h, 72 h, 120 h, 168 h, 240 h, 336 h, 408 h, 504 h, and 672 h post-dose. Serum concentrations of 1B2–10 were then determined with a validated ELISA method. The pharmacokinetic parameters were calculated using the non-compartment analysis (NCA) on WinNonlin Professional software (Certara, Inc., Princeton).

A single-dose toxicity study was conducted on 4 cynomolgus monkeys, divided into two groups (1 animal/sex/group). The animals received either 1B2–10 at 120 mg/kg or a blank lysate control via a single intravenous infusion on day 1. Continuous monitoring was performed 4 hours post-administration, followed by periodic assessments over 28 days for various biochemical parameters, including hemoglobin and hematocrit.

In the 5-week repeated-dose toxicity study, 40 cynomolgus monkeys were randomly assigned to four groups (5 animals/sex/group) and administered doses of 10, 30, or 100 mg/kg once weekly for 5 weeks, followed by a 6-week recovery period. The safety profile of 1B2–10 was evaluated through multiple parameters, including clinical observations, body weight, food consumption, blood pressure, clinical pathology, complete blood count, blood biochemistry, cytokine levels, immunoglobulin concentrations, and serum-specific antibody analyses.

### *In silico* analysis

AlphaFold 3 (35) was employed to predict the structure of 1B2–10 Fv in a complex with hCD47. The predicted complex structure comprises the hCD47 extracellular domain (residues 20-132) and the 1B2–10 Fv, consisting of the heavy chain variable domain (VH) (residues 1-124) and light chain variable domain (VL) (residues 1-106). Following 20 iterations of deep learning with different seeds, the highest-confidence model (seed 10) was selected based on the predicted local distance difference test (pLDDT) score, with interface predicted template modeling (ipTM) and predicted template modeling (pTM) scores of 0.92 and 0.93, respectively.

To further evaluate the accuracy of this complex structure, we performed molecular dynamics (MD) simulations using GROMACS 2024.2. The complex was prepared, solvated, and neutralized, followed by energy minimization and equilibration. MD simulations were conducted for 200 ns at 310 K and 1 bar. Root mean square deviation (RMSD), root mean square fluctuation (RMSF), solvent accessible surface area (SASA), b-factor, and buried surface area (BSA) were analyzed to evaluate the stability and interactions of the antibody-antigen complex throughout the simulation.

The PRODIGY-crystal (36, 37) web server was utilized to explore the interactions between 1B2–10 Fv and CD47. Residue-residue contacts were classified based on their polar, apolar, or charged character and the type of amino acid involved in the contact. A ready-to-run PyMOL script was generated to highlight the interaction interface by displaying and coloring the interacting residues. Visualization and analysis of the complex structure were done in Pymol v3.0.4 (Schrodinger, LLC).

### Statistical analysis

Data are expressed as mean ± standard deviation (SD) or standard error of the mean (SEM). Statistical analyses were performed using GraphPad Prism 8.0 software. Statistical significance was determined by two-way or one-way analysis of variance (ANOVA) as appropriate. Two-way ANOVA was followed by Tukey’s *post hoc* test for multiple comparisons, while ordinary one-way ANOVA was followed by Dunnett’s *post hoc* test for multiple comparisons: *p < 0.05, **p < 0.01, ***p < 0.001, and ****p < 0.0001.

## Results

### The binding affinity and specificity of 1B2–10 to CD47

**Figure 1:**
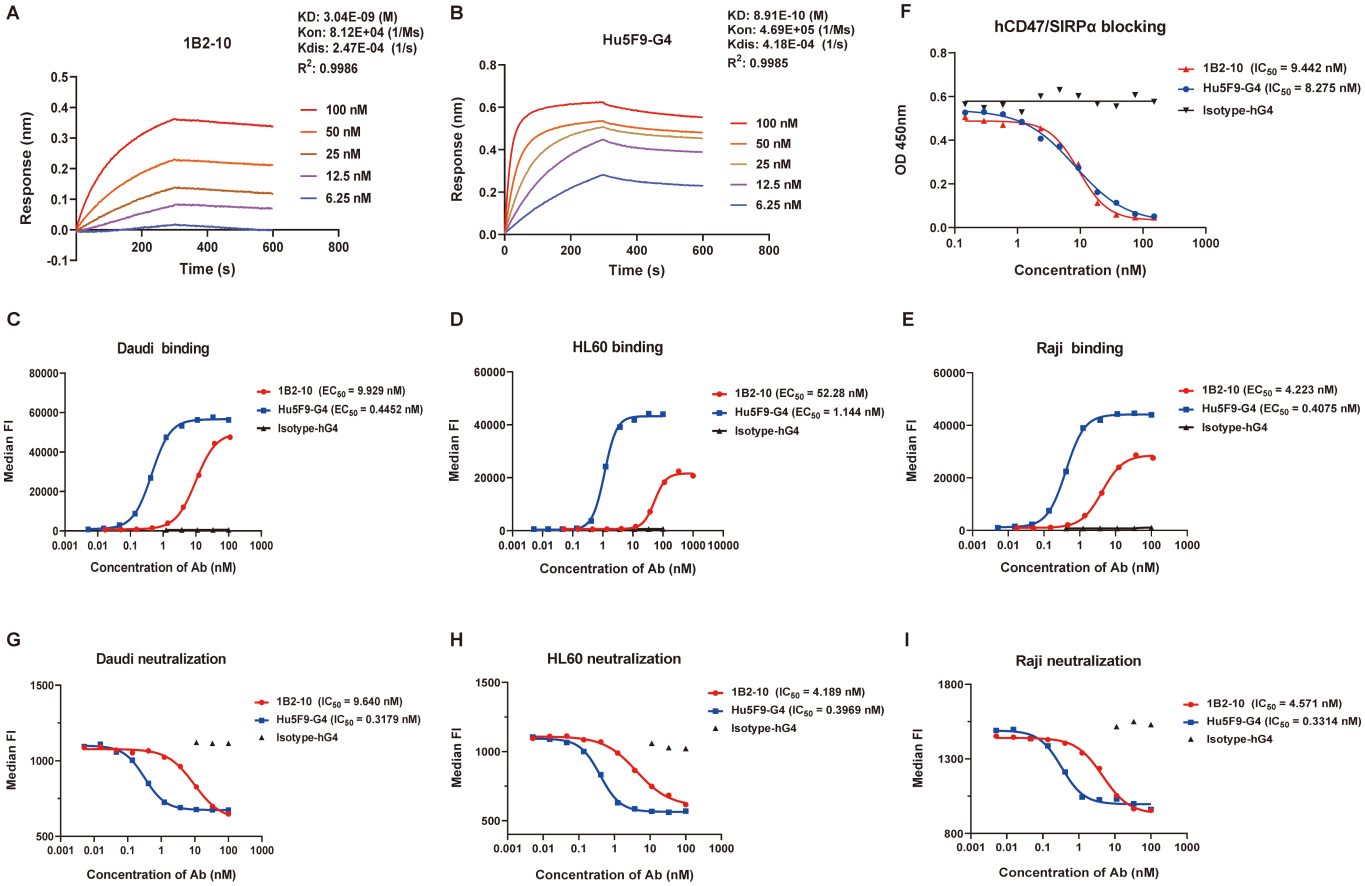
Binding and blocking characteristics of 1B2–10 to human CD47**. (A, B)** Bio-layer interferometry analysis of binding kinetics between human CD47-his and **(A)** 1B2–10 or **(B)** Hu5F9-G4 using a Fortebio Octet 96 system. **(C-E)** Flow cytometric analysis of 1B2–10 binding to CD47 expressed on various tumor cell lines: **(C)** Daudi, **(D)** HL60, and **(E)** Raji. **(F)** Competitive inhibition assay showing 1B2–10 effectively blocking the interaction between hCD47-Fc and recombinant SIRPα-Fc. **(G-I)** Flow cytometric evaluation of 1B2-10-mediated inhibition of recombinant SIRPα binding to CD47 on **(G)** Daudi, **(H)** HL60, and **(I)** Raji cells. All the experiment was performed in twice, and a representative result is presented. Median FI indicates median fluorescence intensity.

The binding affinity and specificity of 1B2–10 to human CD47 (hCD47) were evaluated using ELISA and BLI. ELISA results revealed that 1B2–10 is specifically bound to bivalent hCD47(ECD)-Fc fusion protein with an *EC_50_* value of 0.4551 nM, comparable to that of the reference antibody Hu5F9-G4 (0.2125 nM). 1B2–10 also bound to monovalent hCD47 ECD- his fusion protein with an *EC_50_* value of 2.0 nM (data not shown). Furthermore, 1B2–10 showed binding to cynomolgus monkey CD47 ECD-Fc fusion protein (**Supplementary Figures S1A-B**) but did not react with mouse or rat CD47-Fc. BLI analysis determined the equilibrium dissociation constant (*KD*) of 1B2–10 to be 3.04 nM, compared to 0.89 nM for Hu5F9-G4 (**Figures 1A, B**). Flow cytometry analysis showed that 1B2–10 bound to lymphoma Daudi, HL60, and Raji cells with an *EC_50_* of 9.93 nM, 52.28 nM and 4.223 nM, respectively (**Figures 1C-E**). Additional tumor cell lines, including A549, SKBR-3, and HT-29 cells, were also tested, with *EC_50_* of 41.49 nM, 29.56 nM and 44.80 nM, respectively (**Supplementary Figures S1C-E**). Furthermore, 1B2–10 displayed a considerably lower binding affinity to CD3^+^ T cells (*EC_50_* = 151.5 nM) and CD19^+^ B cells (*EC_50_* = 49.77 nM) compared to Hu5F9-G4 (*EC_50_* = 11.80 nM and 1.593 nM, respectively) (**Supplementary Figures S1F-G**), indicating its potentially reduced off-target effects.

### 1B2–10 neutralized CD47/SIRPα interaction

Competitive ELISA results demonstrated that 1B2–10 inhibited the binding of hCD47 ECD-Fc to hSIRPα ECD-Fc fusion protein with an *IC_50_* value of 9.44 nM, which was comparable to that of Hu5F9-G4 (*IC_50_* = 8.28 nM) when hCD47 ECD-Fc used at 50 nM (**Figure 1F**). Furthermore, 1B2–10 potently inhibited the binding of recombinant hSIRPα ECD-Fc fusion protein (used at 18 nM) to Daudi, HL60, and Raji cells, with *IC_50_* values of 9.64 nM, 4.19 nM, and 4.57 nM, respectively. The inhibitory potency of 1B2–10 on hSIRPα-Fc binding to tumor cells was approximately 10-fold lower than that of Hu5F9-G4 (*IC_50_* values of 0.32 nM, 0.40 nM, and 0.33 nM) for the respective cell lines (**Figures 1G-I**).

### 1B2–10 showed minimal erythrocyte and thrombocyte binding and no hemagglutination

**Figure 2:**
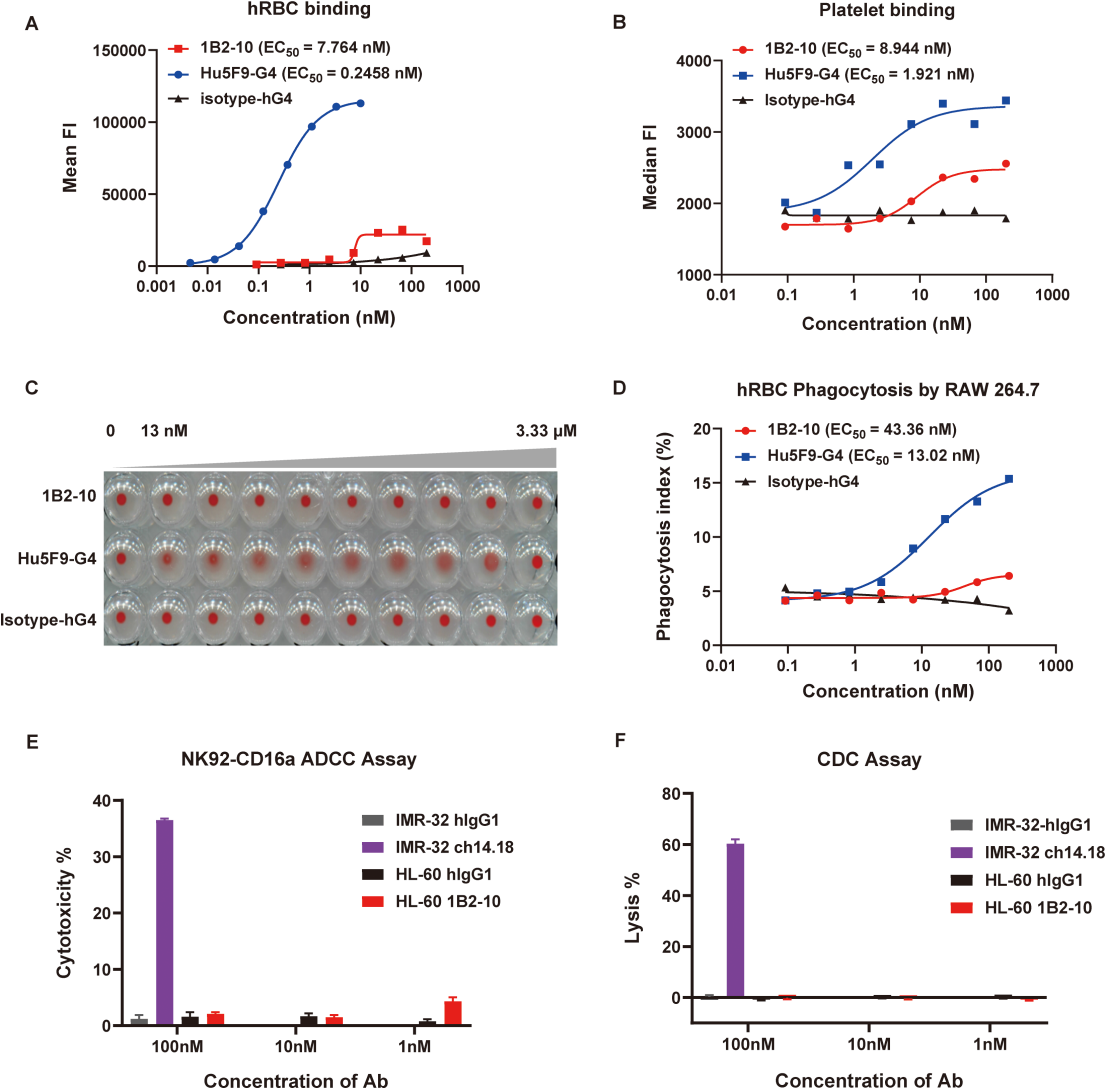
Safety evaluation of 1B2-10. **(A)** and **(B)** Flow cytometric analysis of 1B2–10 and Hu5F9-G4 binding to human red blood cells (hRBCs) **(A)** and human thrombocytes **(B)**. **(C)** Hemagglutination activity of 1B2-10, Hu5F9-G4, or isotype control on human erythrocytes following 2-hour incubation at 37°C. **(D)** Quantification of macrophage-mediated phagocytosis of erythrocytes. The phagocytosis index was determined by flow cytometric analysis. **(E)** and **(F)** ADCC and CDC effects of 1B2–10 on HL-60 target cells. For the ADCC assay, NK-CD16a cells were used as effectors with an effector-to-target ratio (NK-CD16a: HL-60) of 5:1. The cell densities were 3×10^6^ cells/mL for NK-CD16a and 6×10^5^ cells/mL for HL-60. In the CDC assay, HL-60 cells were maintained at 6×10^5^ cells/mL. Anti-GD2 antibody ch14.18 was a positive control, with IMR-32 as the target cell, hIgG1 isotype antibody was used as a negative control. The above experiments were performed in triplicate, and data were presented as mean ± SD.

We compared the binding affinities of 1B2–10 and Hu5F9-G4 to erythrocytes and thrombocytes using flow cytometry. 1B2–10 demonstrated minimal binding to both human (**Figure 2A**) and cynomolgus monkey erythrocytes (**Supplementary Figure S2A**), whereas Hu5F9-G4 showed robust binding affinity. Moreover, 1B2–10 exhibited limited binding to human thrombocytes with an *EC_50_* value of 1.92 nM, in contrast to the stronger binding observed for Hu5F9-G4 (*EC_50_* value of 8.94 nM) (**Figure 2B**). Significantly, 1B2–10 did not induce agglutination of human (**Figures 2C**, **S2B**) or cynomolgus monkey erythrocytes (**Supplementary Figures S2C-D**) even at concentrations up to 3.3 μM. In contrast, Hu5F9-G4 induced hemagglutination at concentrations as low as 13 nM. Additionally, erythrocyte phagocytosis assays demonstrated that 1B2–10 induced negligible phagocytic activity compared to Hu5F9-G4 (**Figure 2D**). To further evaluate the antibody-mediated cytotoxicities of 1B2-10, ADCC and CDC experiments were conducted using HL-60 cells as targets. The results demonstrated that while positive control antibody ch14.18 exhibited a typical dose-response activity, 1B2–10 showed no detectable ADCC or CDC activity across the concentration range tested (**Figures 2E, F**). These findings collectively suggest a favorable safety profile for 1B2–10 in both preclinical and clinical studies.

### 1B2–10 promoted phagocytosis of tumor cells *in vitro*

**Figure 3:**
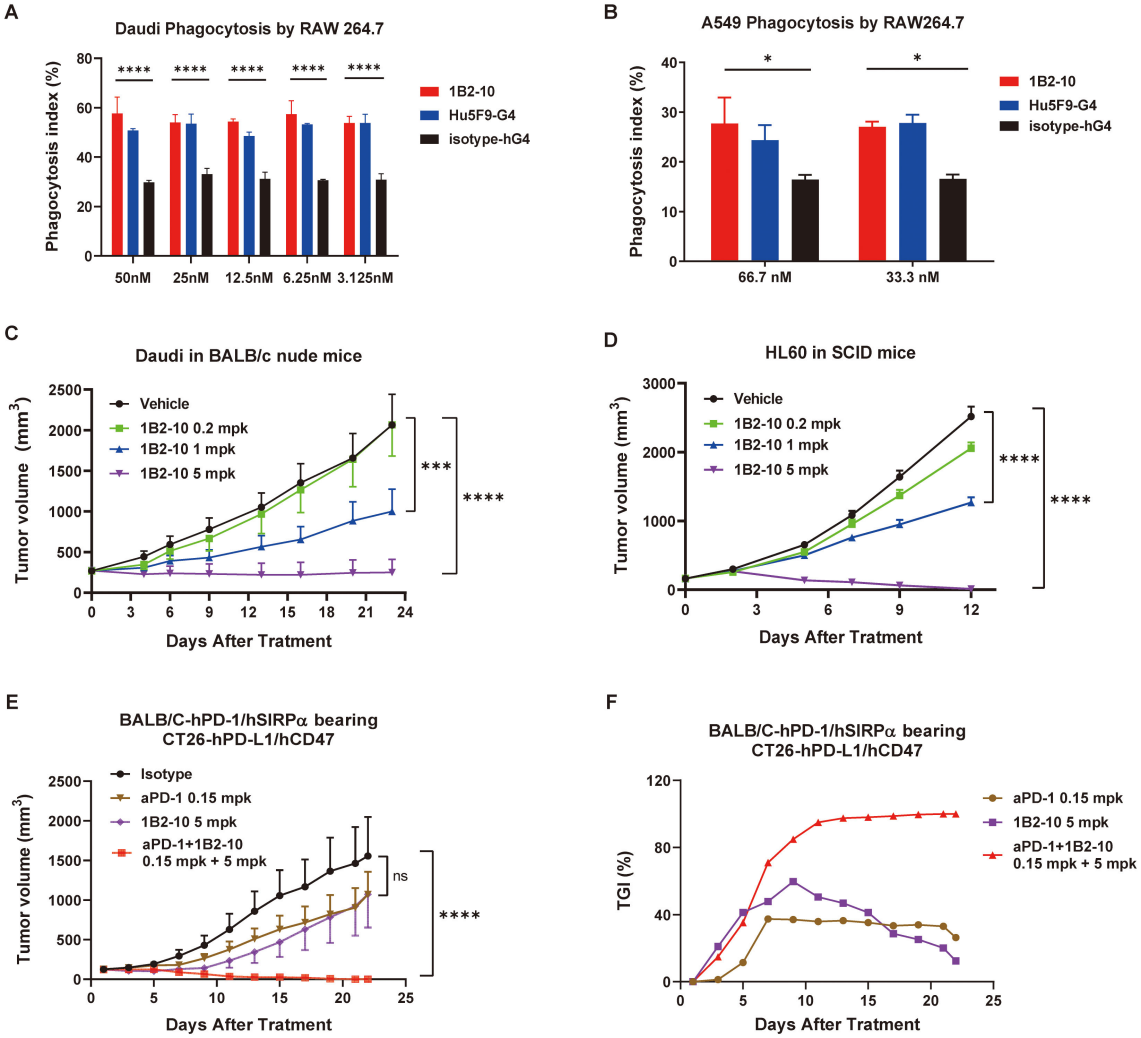
1B2–10 enhances macrophage-mediated phagocytosis of tumor cells and demonstrates potent anti-tumor activity in monotherapy and combination therapy. **(A)** and **(B)** Quantification of macrophage-mediated phagocytosis of human Daudi cells **(A)** and A549 cells **(B)**. The phagocytosis index was determined by flow cytometric analysis. Experiments were performed in duplicate, and data are presented as mean ± SD. **(C, D)** BALB/c nude mice (C, n=10/group) and SCID/Beige mice (D, n=10/group) were subcutaneously inoculated with lymphoma Daudi and HL-60 cells, respectively. Upon reaching a tumor volume of 100–200 mm^3^, mice were administered 1B2–10 at varying doses (0.2, 1.0, and 5.0 mg/kg) or vehicle control. Tumor volumes were assessed thrice weekly and are presented as mean tumor volume ± SEM. **(E, F)** Growth curves of CT26-hPD-L1/hCD47 tumors (E, n=10/group) in BALB/c-hPD-1/hSIRPα mice and corresponding tumor growth inhibition (TGI) **(F)** following treatment with indicated doses of 1B2–10 alone or in combination with anti-PD-1 antibody. (Statistical analysis: Two-way ANOVA with Tukey’s multiple comparisons tests; ns: not significant, *p < 0.05, ***p < 0.001, ****p < 0.0001).

Phagocytosis assays were performed using a mouse macrophage-like cell line RAW264.7 cells as effector cells, while Daudi and A549 tumor cells as target cells. RAW 264.7 cells were fluorescently labeled with cell proliferation Dye eFluor™ 670 and then co-cultured with CFSE-positive Daudi lymphoma cells in the presence of either CD47 antibodies or an IgG4 isotype control. The phagocytosis index was calculated to quantitatively assess the extent of phagocytosis. Notably, 1B2–10 demonstrated robust phagocytosis induction across a concentration range of 3.12 nM to 50 nM, exhibiting efficacy comparable to that of Hu5F9-G4 (**Figure 3A**). This enhanced phagocytic activity was further corroborated using A549 cells as target cells (**Figure 3B**). These findings collectively underscore the potent ability of 1B2–10 to augment macrophage-mediated phagocytosis, suggesting a potential mechanism underlying its observed anti-tumor activity.

### 1B2–10 inhibited tumor growth as a monotherapy

The anti-tumor effects of 1B2–10 were investigated in tumor xenograft mouse models inoculated with Daudi and HL-60 cells. The results demonstrated that tumor growth in both Daudi and HL-60 xenograft models was significantly inhibited in a dose-dependent manner (**Figures 3C, D**). In Daudi xenografts, TGI values of 87.92% and 51.57% were observed at 5 and 1 mg/kg, respectively. Similarly, in HL-60 xenografts, TGI values of 99.52% and 50.17% were recorded at the same doses. No significant tumor-suppressive effect was observed at 0.2 mg/kg in either model (**Supplementary Figures S3A, S3C**). Importantly, the majority of 1B2-10-, but not isotype-treated, animals maintained their body weights throughout the study (**Supplementary Figures S3B, S3D, and S3E**).

### 1B2–10 inhibited tumor growth in combination with PD-1 antibody

The CT26-hPDL1/hCD47 syngeneic tumor model was established in hPD-1/hSIRPα BALB/c mice. In this model, either 1B2–10 or a PD-1 antibody monotherapy alone showed inhibition on tumor growth, with Peak TGI values of 59.73% and 37.36%, respectively. Remarkably, treatment with 1B2–10 in combination with PD-1 antibody completely abolished the tumor growth (**Figures 3E, F**). 1B2–10 caused no significant changes in body weight (**Supplementary Figure S3F**). These findings indicated that 1B2–10 can act synergistically with PD-1/PD-L1 pathway inhibitors in cancer immunotherapies.

### 1B2–10 had a favorable safety profile in cynomolgus monkeys

Toxicity assessments of 1B2–10 were performed in cynomolgus monkeys following a single or 5-week consecutive repeat-dose toxicity study (QW × 5) with a 6-week recovery period. In the single-dose toxicity study, two groups of cynomolgus monkeys were administered 1B2–10 at doses of 0 and 120 mg/kg via intravenous injection. Clinical signs, body weight, food consumption, ECG, and hematological parameters were evaluated in all animals during the 28-day observation period. No significant hematological adverse effects were observed in either male or female cynomolgus monkeys. As shown in **Supplementary Figures S4A-D**, 1B2–10 treatment was well-tolerated with no statistically significant difference observed between the vehicle control and 1B2-10-treated groups at doses up to 120 mg/kg for erythrocyte counts, hemoglobin (HGB) concentration, the percentage of hematocrit (HCT), and thrombocyte (PLTs) levels. The no-observed adverse-effect level (NOAEL) was determined to be 120 mg/kg.

**Figure 4:**
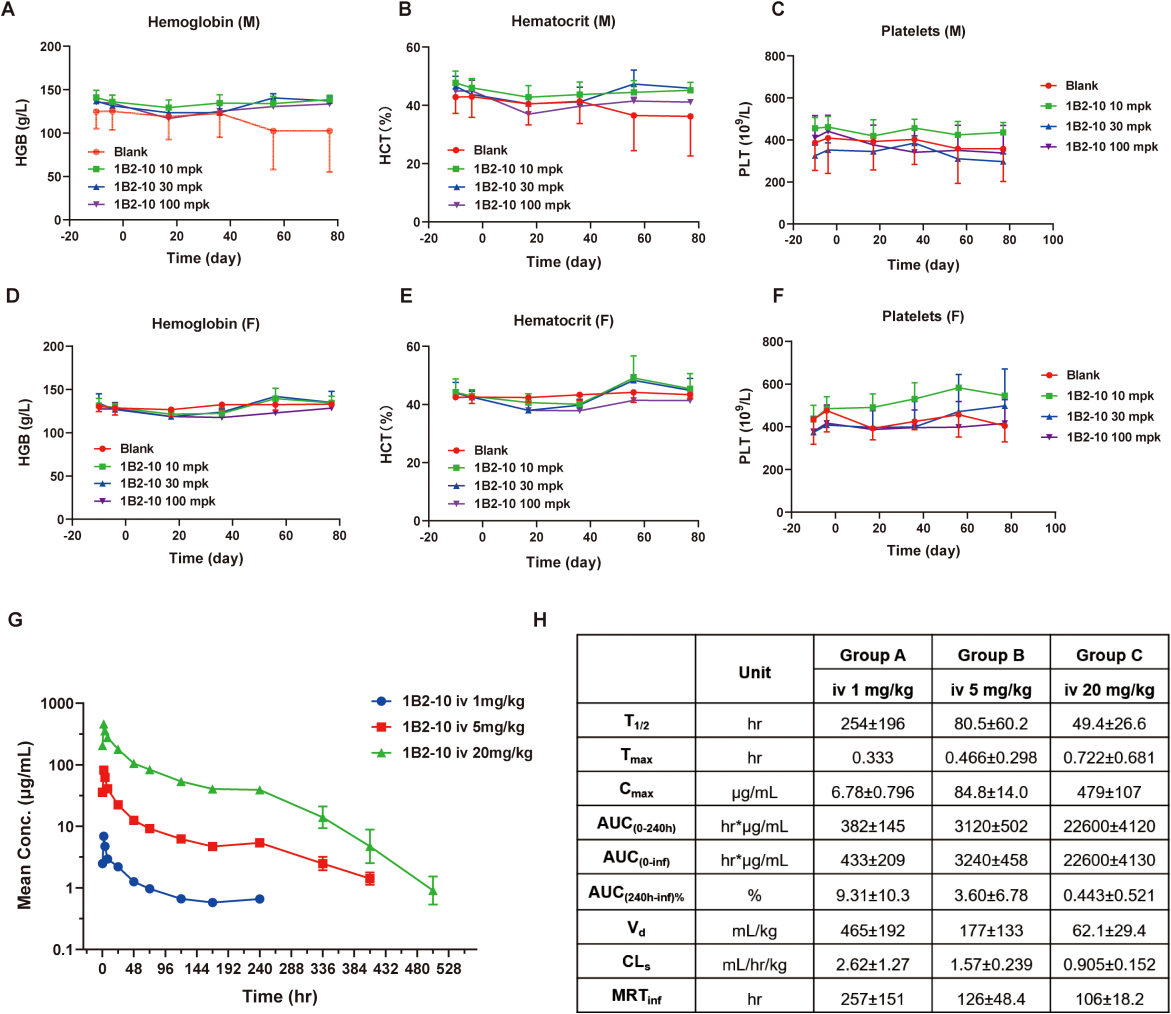
Pharmacokinetic and toxicology studies of 1B2–10 in non-human primates. **(A, D)** Changes in hemoglobin (HGB) levels in response to treatment with different doses of 1B2–10 in male (M) and female (F) monkeys. **(B, E)** Changes in hematocrit (HCT) percentage in response to treatment with different doses of 1B2–10 in male (M) and female (F) monkeys. **(C, F)** Changes in thrombocyte (PLT) counts in response to treatment with different doses of 1B2–10 in male (M) and female (F) monkeys. **(G)** Mean pharmacokinetic profile of 1B2–10 in cynomolgus macaques. Single doses of 1B2-10 (1, 5, and 20 mg/kg) were administered intravenously to cynomolgus monkeys, and serum samples were collected and analyzed at various time points. **(H)** Pharmacokinetic parameters derived from panel **(G)**. All data are presented as mean ± SD.

In a follow-up study, repeated intravenous injections of 1B2–10 were administrated to cynomolgus monkeys at 10, 30, or 100 mg/kg once weekly for 5 weeks. All dose levels were clinically tolerated, with no notable clinical signs or effects on appearance observation, body weight, or food consumption. Levels of HGB, HCT, and PLTs (**Figures 4A-F**) were rarely found to be significantly reduced compared to formulation buffer-treated monkeys, and no anemia or thrombopenia was observed in monkeys during the observing period. No sex-related differences in exposure and no accumulation of 1B2–10 were detected in the repeated dose toxicity study. The NOAEL was determined to be 100 mg/kg/dose for up to five weekly doses.

### Pharmacokinetics profile of 1B2–10 in cynomolgus monkeys

The pharmacokinetics profile of 1B2–10 was evaluated in cynomolgus monkeys following a single intravenous infusion of 1B2–10 at doses of 1, 5, or 20 mg/kg (**Figure 4G**). No consistent or significant sex-related differences in systemic exposure were observed. Based on mean *Cmax* and *AUC_0–240 h_* values, systemic exposure to 1B2–10 increased in a greater than dose-proportional manner across the 1 to 20 mg/kg dose range in both sexs. Across dose levels, the average elimination half-life (*T_1/2_*) ranged from 49.4 to 254 h, *Cmax* from 6.78 to 479 μg/mL, *AUC_0–240 h_* from 382 to 22600 h*μg/mL, volume of distribution (*Vd*) from 465 to 62.1 mL/kg, clearance (*CL*) from 2.62 to 0.905 mL/h/kg, and mean residence time (*MRT_inf_*) from 106 to 257 h (**Figure 4H**). These data indicate that 1B2–10 was distributed beyond the circulatory system. Moreover, the *Vd* approached plasma volume after target saturation, which is similar to the characteristic of intravenously administered mAb therapeutics.

### *In silico* analyses of binding epitope

To explore the molecular basis by which 1B2–10 differentially recognizes tumor cells from erythrocytes, we analyze the epitope of CD47 interacting with 1B2-10. AlphaFold 3 predicted a high-confidence 1:1 complex structure of 1B2–10 Fv bound to the human CD47 ECD, yielding impressive ipTM and pTM scores of 0.92 and 0.93, respectively. Subsequent molecular dynamics simulations corroborated the stability of this interaction, revealing that the interface RMSD (I-RMSD) of the predicted structure stabilized at approximately 0.2-0.3 nm (**Supplementary Figure S5A**), while most of the RMSF values converged to 0.1-0.3 nm (data not shown). The fluctuations in the number of hydrogen bonds (H-bonds) between the donor (CD47) and acceptor (1B2-10) atoms were within a defined range (**Supplementary Figure S5C**), indicating a well-equilibrated hydrogen bond network at the antigen-antibody interface throughout the majority of the simulation. These results collectively suggest high confidence in the predicted structure and demonstrate robust, stable binding between hCD47 and 1B2–10 Fv.

**Figure 5:**
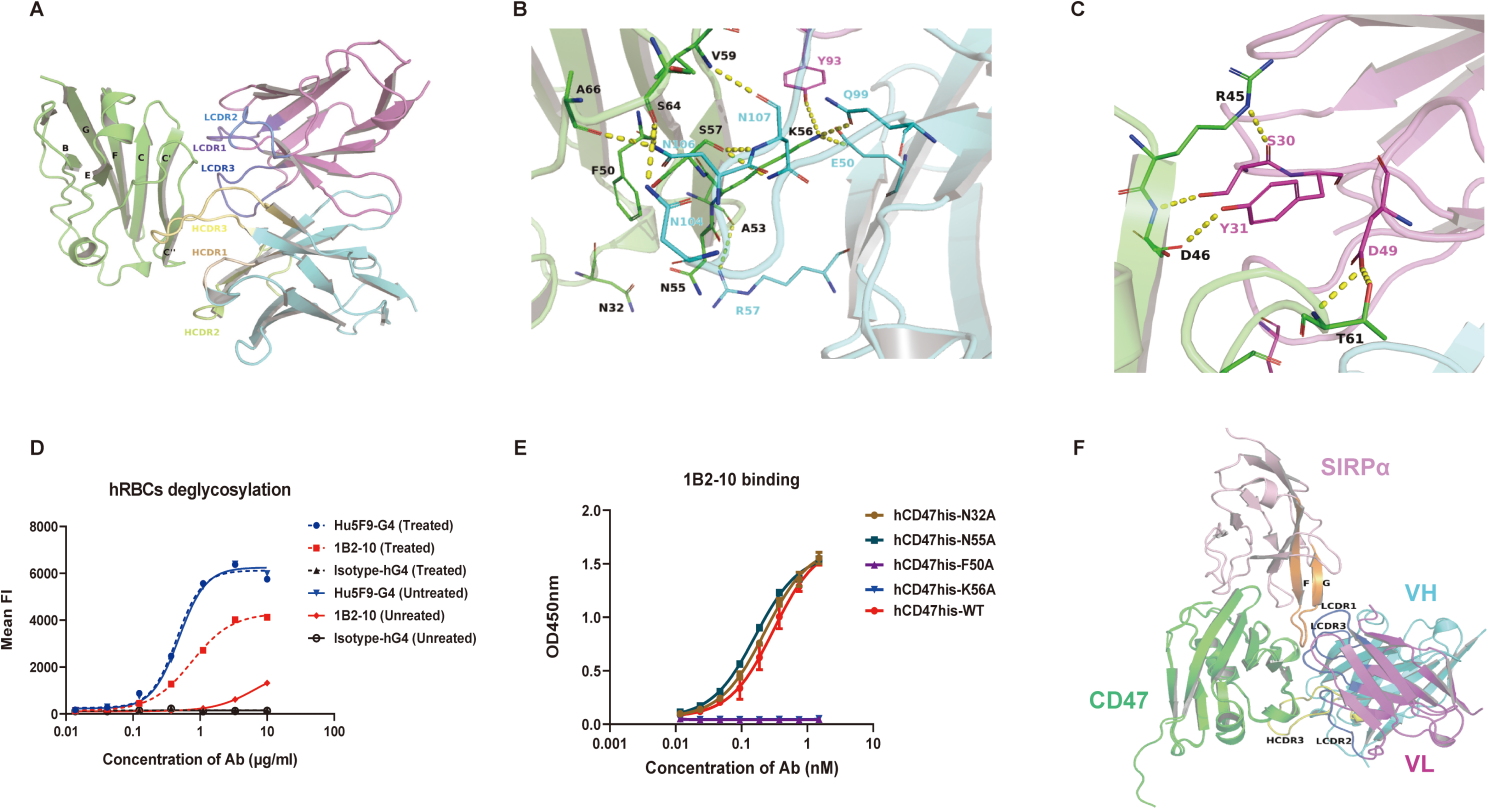
In silico and mutagenesis analysis of the interaction between 1B2–10 Fv and hCD47-ECD. **(A)** Overall structure and interaction of 1B2–10 Fv in complex with hCD47-ECD. The interaction between CDR regions of VH and VL of 1B2–10 Fv with the C, C’, C’’ strands, CC’ loop, and C’C’’ loop of CD47 is shown. **(B)** Polar interactions of VH CDR2, CDR3, and VL CDR3 with CD47. Detailed residue interactions of 1B2–10 Fv with hCD47 are depicted. **(C)** Polar interactions of VL CDR1 and CDR2 with CD47. CD47 is shown as a green cartoon, while cyan and magenta represent the VH and VL of 1B2–10 Fv, respectively. Residues involved in the interaction are shown as sticks and polar interactions are indicated by dashed yellow lines. Residues on CD47 are colored black, while residues on VH and VL of 1B2–10 Fv are colored cyan and magenta, respectively. **(D)** 1B2–10 exhibited increased binding to deglycosylated human erythrocytes treated with PNGase F overnight, as analyzed by flow cytometry. **(E)** ELISA analysis of 1B2–10 binding to alanine mutants of human CD47-His protein. **(F)** Superimposition of CD47 from the CD47/SIRPα (V1) complex (PDB ID: 4CMM) with CD47 from the 1B2–10 Fv/CD47 complex predicted by AlphaFold 3. Green represents CD47, and pink represents SIRPα.

The binding interface analysis revealed substantial interactions between 1B2–10 Fv and specific regions of CD47, including the C, C’, and C’’ strands, CC’ and C’C’’ loops, and the α-helix proximal to the C’’ strand. All three complementarity-determining regions (CDRs) of both the heavy chain (VH) and light chain (VL) contribute to the interface with hCD47-ECD (**Figure 5A**). The amino acid residues involved in polar interactions are illustrated in **Figures 5B, C**. Human CD47-ECD harbors five potential N-linked glycosylation sites at N5, N16, N32, N55, and N93. Notably, our analyses identified the involvement of N-linked glycans at positions N32 and N55 of hCD47 in modulating 1B2-10-Fv binding. The N55 glycan, in particular, extends toward the core interface and may influence 1B2–10 binding affinity. Furthermore, our observations revealed that the highly flexible EAQNTT loop (residues 29–34 of hCD47-ECD) exhibited significant structural fluctuations during the 150–200 ns period of molecular dynamics simulations (**Supplementary Figures S5A, B**). This led to an increase in the contact area between 1B2–10 Fv and CD47, underscoring the importance of this region for CD47 binding. The N32 glycosylation site was also involved in this process (**Supplementary Figure S5D-E**). These findings were corroborated by the enhanced 1B2–10 binding observed following deglycosylation of erythrocytes via PNGase treatment, confirming that N-linked glycosylation of CD47 contributes to the lower binding affinity of 1B2–10 to erythrocytes (**Figure 5D**).

To further validate the predicated 1B2–10 binding epitope, we generated four human CD47-His protein mutants: N32A, N55A, F50A, and K56A. Based on our structural model, we selected N32 and N55 as glycosylation sites proximal to the predicted core binding site, and F50 and K56 as residues within the predicted binding site’s hydrophobic core. All four mutants exhibited comparable binding to SIRPα, confirming their structural integrity and biological activity. Subsequent ELISA analysis (**Figure 5E**) revealed that alanine substitution of F50 and K56 (F50A and K56A) completely abolished 1B2–10 binding. This finding strongly indicates that F50 and K56 are critical residues for 1B2–10 recognition, consistent with our structural predictions. Interestingly, mutation of the glycosylation sites (N32A and N55A) did not affect 1B2–10 binding, a result that contrasts with our observation that deglycosylation of erythrocytes significantly enhances 1B2–10 binding. This discrepancy likely stems from variations in CD47 glycosylation patterns between HEK293F cells (used for CD47-His protein production) and erythrocytes. We hypothesize that CD47 protein produced in HEK293F cells exhibits a lower degree of glycosylation, such that mutations at N32 and N55, despite their proximal to the core epitope, have a minimal effect on 1B2–10 binding. Conversely, the more extensive glycosylation on erythrocytes may result in glycan moieties near the core epitope (N32 and N55) contributing to steric hindrance of 1B2–10 binding, an effect that is alleviated upon deglycosylation. Further, more sophisticated structural analyses are needed to fully elucidate this mechanism.

In comparison to the complex structure of CD47/SIRPα (PDB code: 4CMM), 1B2–10 Fv exhibits a distinct binding pattern to CD47. Unlike other reported CD47-blocking antibodies that primarily target the FG loop of CD47, which serves as a blocking “hotspot” (38), 1B2–10 Fv binds to the BC loop and C/C’ strand (**Figures 5F**, **S5F**). This binding site coincides with the SIRPα binding surface, indicating that 1B2–10 directly competes with SIRPα for CD47 binding through a novel epitope.

## Discussion

The therapeutic potentials of CD47-neutralizing antibodies have been significantly compromised by dose-limiting hematologic toxicities and target-mediated rapid clearance, primarily due to the ubiquitous expression of CD47 on normal cells, especially erythrocytes, and thrombocytes. The early CD47-blocking mAb Hu5F9-G4 exhibited a high affinity for CD47 on erythrocytes and thrombocytes and encountered significant challenges in clinical development due to severe anemia and thrombopenia. Despite subsequent mAb iterations attempting to mitigate hemagglutination, significant safety concerns persist in effectively addressing hematological toxicities.

Herein, we report the development of 1B2-10 (also named DS003), a CD47-neutralizing mAb with an improved efficacy/safety ratio over previous therapeutics. Our *in vitro* studies demonstrated that 1B2–10 effectively disrupted the interaction between CD47 and SIRPα, thereby abrogating the “don’t eat me” signal and resulting in a significant enhancement of macrophage-mediated phagocytosis against CD47-expressing cancer cell lines. Notably, 1B2–10 achieves its therapeutic effects without inducing ADCC or CDC, potentially minimizing the off-target effects (**Figures 2E, F**). Unlike Hu5F9-G4, 1B2–10 demonstrated a negligible binding affinity for erythrocytes and there was no detectable erythrocyte aggregation at equivalent concentrations. Furthermore, 1B2–10 exhibited negligible erythrophagocytosis, whereas a CD47 antibody AK117 still induced significant phagocytosis of erythrocytes. This enhanced clearance of erythrocytes by macrophages appeared to be the primary mechanism underlying the transient anemia observed with AK117 in cynomolgus monkeys (21). Additionally, 1B2–10 demonstrated minimal thrombocyte interaction, substantially reducing the probability of macrophage-mediated thrombocyte phagocytosis and by extension, thrombopenia. Notably, thrombocyte binding or phagocytosis, the primary mechanisms underlying thrombopenia, have not been investigated for most CD47 antibodies in preclinical studies. AO-176 exhibited reduced, but still substantial thrombocyte binding compared to tumor cells (24). These collective characteristics confer a favorable hematological safety profile for 1B2–10 *in vitro*, representing a significant advancement of CD47-neutralizing mAbs in efficacy/safety ratio.

In tumor xenograft models, 1B2–10 as monotherapy demonstrated a minimum effective dose (MED) of 1 mg/kg, twice better than that of TJC4, which was determined to be 2 mg/kg (27). Besides blocking the “don’t eat me” signal, however, it is also crucial to concurrently enhance the “eat me” signals to maximize the phagocytic efficiency of macrophages (6, 39, 40). Consequently, CD47-blocking antibodies are being explored in combination with various antineoplastic agents, including chemotherapy drugs such as azacytidine, and antibodies that activate FcR or modulate adaptive immunity. These combinational strategies aim to synergistically augment phagocytosis and elicit robust immunological responses that promote both the innate and adaptive antitumor mechanisms. Several clinical studies have demonstrated that combination approaches targeting both PD-1 (or PD-L1) and CD47, as bispecific molecules or combination therapies, increased efficacy and safety ratios (41, 42). Compared to either agent alone, 1B2–10 combined with a PD-1 antibody showed significant enhancement in their antitumor effects. Moreover, 1B2–10 displayed encouraging safety profiles in monkeys, demonstrating good tolerability at the investigated doses.

In cynomolgus monkeys, 1B2–10 demonstrated an exceptional safety profile. Following single-dose administration, negligible hematotoxicities were observed with an MTD as high as 120 mg/kg. Similarly, in the one-month repeated-dose toxicity study, no significant hematotoxicities were observed, and the NOAEL was as high as 100 mg/kg, which was significantly higher than the clinical doses of 10–30 mg/kg for most CD47 antagonists (20, 43, 44). Pharmacokinetic studies revealed dose-proportional increases in serum concentrations, consistent with the absence of a saturable CD47 antigen “sink”. However, unexpected dose-dependent elimination kinetics were observed (**Figures 4G, H**), with the 1 mg/kg dose exhibiting a substantially longer half-life (~250 hours) compared to 5 mg/kg and 20 mg/kg doses (81 and 49 hours, respectively). This inverse dose-half-life relationship suggests target-mediated drug disposition (TMDD) mechanisms within the circulation. This phenomenon likely reflects concentration-dependent binding to CD47-expressing blood cells. At low concentrations (1 mg/kg), predominantly linear elimination results in prolonged circulation. Conversely, at higher concentrations (5, 20 mg/kg), enhanced binding to circulating blood cells facilitates accelerated clearance via the reticuloendothelial system. The rapid decline in drug exposure after day 10 suggests anti-drug antibody (ADA) development, which can compromise exposure through immune complex formation and enhanced clearance. Evaluation of ADA status in study animals would be essential to confirm this hypothesis. Despite these complex pharmacokinetic behaviors, the overall linear dose-exposure relationship supports a favorable safety profile. These insights provide valuable guidance for optimal clinical dosing strategies. Collectively, these findings demonstrate a promising safety profile for 1B2-10 (DS003), currently under Phase I clinical evaluation with comprehensive efficacy and immunogenicity monitoring.

Erythrocytes are characterized by a high degree of membrane protein glycosylation. It was reported previously that some SIRPα mutants and CD47 antibodies exhibit differential bindings to CD47 on malignant and normal cells, due possibly to distinct glycosylation patterns of CD47 molecule (45). Molecular simulation-based spatial structure analyses and mutagenesis experiment revealed that two N-linked glycosylation sites, proximity to a critical binding epitope of 1B2-10, may contribute significantly to its tumor cell selectivity. A similar mechanism was proposed for TJC4, whose binding to erythrocytes was occluded by an N-linked glycan that functions as a “shield” (27). 1B2–10 exhibited a distinctive mechanism of action by directly inhibiting CD47-SIRPα interaction through the C/C’/C’’ strand, in contrast to previously reported CD47-blocking agents that primarily target the FG-loop-dominated “hotspot” (38). Structural analysis reveals that the C/C’/C’’ strand region represents a less conserved binding interface compared to the traditional FG-loop hotspot, potentially explaining the differential binding profiles observed between tumor cells and normal erythrocytes. This alternative binding site may be subject to distinct glycosylation patterns and conformational changes that favor selective recognition of tumor cells over healthy cells. This unique blocking strategy represents a distinct approach for designing anti-CD47 antagonists with reduced erythrocyte binding, offering the potential to maintain robust antitumor efficacy while minimizing hematological toxicities.

Despite several setbacks in clinical trials due to limited monotherapy efficacy and hematological toxicities, CD47-targeted therapies remain promising. Currently, over 10 CD47-related therapeutic agents are undergoing evaluation in more than 40 clinical trials worldwide, targeting various hematological malignancies as well as solid tumors. The suboptimal efficacy of CD47-targeting monotherapy can be attributed to multiple factors, including the complexity of the tumor microenvironments, cancer cell heterogeneity, immune system dysfunction and modulation, and off-target effects, but the utmost factor has been hematotoxicity, which ultimately set the upper limit of the therapeutic doses (15). Beyond attempts to reduce hematotoxicity, researchers are exploring diverse strategies to enhance therapeutic efficacy through multi-modal treatments, which have comprised over 90% of ongoing clinical studies. These approaches include bsAbs, dual immune checkpoint inhibitors (ICIs), dual chimeric antigen receptor T (CAR-T) cells, and other innovative combinations (46–48). In these treatment regimens, CD47-targeted agents, playing either major or supportive roles, demonstrated superiority to conventional therapies, particularly in the treatment of acute myeloid leukemia (AML). The incorporation of CD47-targeted agents has shown promise in enhancing the efficacy of existing treatments or overcoming drug resistance. While preliminary results from some of these approaches are encouraging, further comprehensive research is essential to optimize CD47-involving combination strategies, with the dual objectives of maximizing therapeutic efficacy and minimizing toxicity. Moving forward, the development of CD47-targeted therapeutics will continue focusing on addressing safety concerns while maximizing the therapeutic potential.

This study is subject to several limitations. First, our *in vitro* phagocytosis assay employed a co-culture system of murine macrophages and human cells. This approach may have introduced non-species specific CD47/SIRPα interactions; therefore, a fully human macrophage-based system is necessary to comprehensively evaluate antibody functionality *in vitro*. Second, the current preclinical evaluation relies primarily on xenograft models using human tumor cell lines and syngeneic models employing modified cell lines, which are insufficient to fully recapitulate immune microenvironment-mediated mechanisms of tumor inhibition. Moreover, the exclusive use of female mice limits the translational relevance of our findings. Future studies will incorporate both sexes to improve translational fidelity. The implementation of primary patient-derived xenografts or humanized mouse models is warranted for a more rigorous preclinical assessment. Finally, the structural specificity rationale, currently supported by *in silico* analyses and mutagenesis experiments, will be further substantiated through high-resolution structural biology techniques, including X-ray crystallography, hydrogen-deuterium exchange mass spectrometry, and comprehensive glycosylation profiling.

## Conclusions

Our investigation underscores the promise of 1B2–10 as a promising therapeutic anti-CD47 mAb. This agent has demonstrated the capability to elicit antitumor activity through macrophage-mediated phagocytosis while minimizing the hematological toxicities. These characteristics of 1B2–10 suggest improved safety and substantial therapeutic potential for cancer patients, both as a single agent and in combination with other antitumor therapies.

## Data Availability

The datasets supporting the findings of this study are available from Shanghai Mabstone Biotechnologies under license and are not publicly available due to restrictions. However, all research data generated during the current study are available from corresponding author upon reasonable request.
