# Supplementary material for: A CD47 antibody with minimized erythrocyte and thrombocyte toxicities
Source: Front Oncol. 2025 Nov 26;15:1686180. doi: 10.3389/fonc.2025.1686180 (PMC12690563; doi:10.3389/fonc.2025.1686180)
Supplement: Supplementary file 1 [file DataSheet1.docx]

**Supplementary Materials**


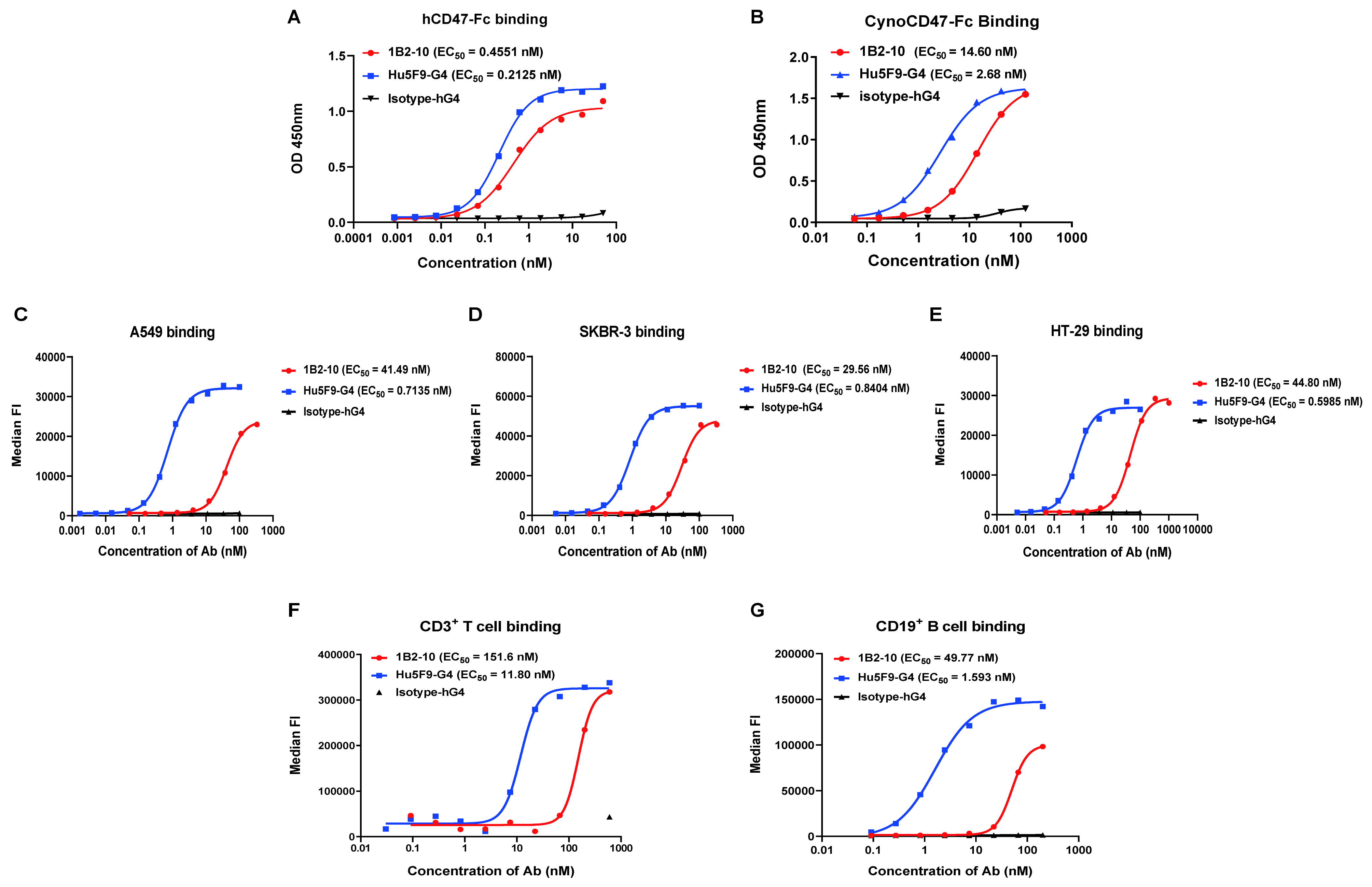


**Figure S1** Cross-species binding affinity of 1B2-10 to hCD47 and its interaction with tumor cell lines. **(A)** and **(B)** ELISA-based quantification of 1B2-10 binding to (A) human and (B) cynomolgus monkey CD47-Fc. **(C-E)** Flow cytometric analysis of 1B2-10 binding to CD47 expressed on human tumor cell lines: (D) A549, (E) SKBR-3, and (F) HT-29. **(F-G)** Flow cytometric analysis of 1B2-10 binding to CD47 expressed on human CD3^+^ T cell and CD19^+^ B immune cells.


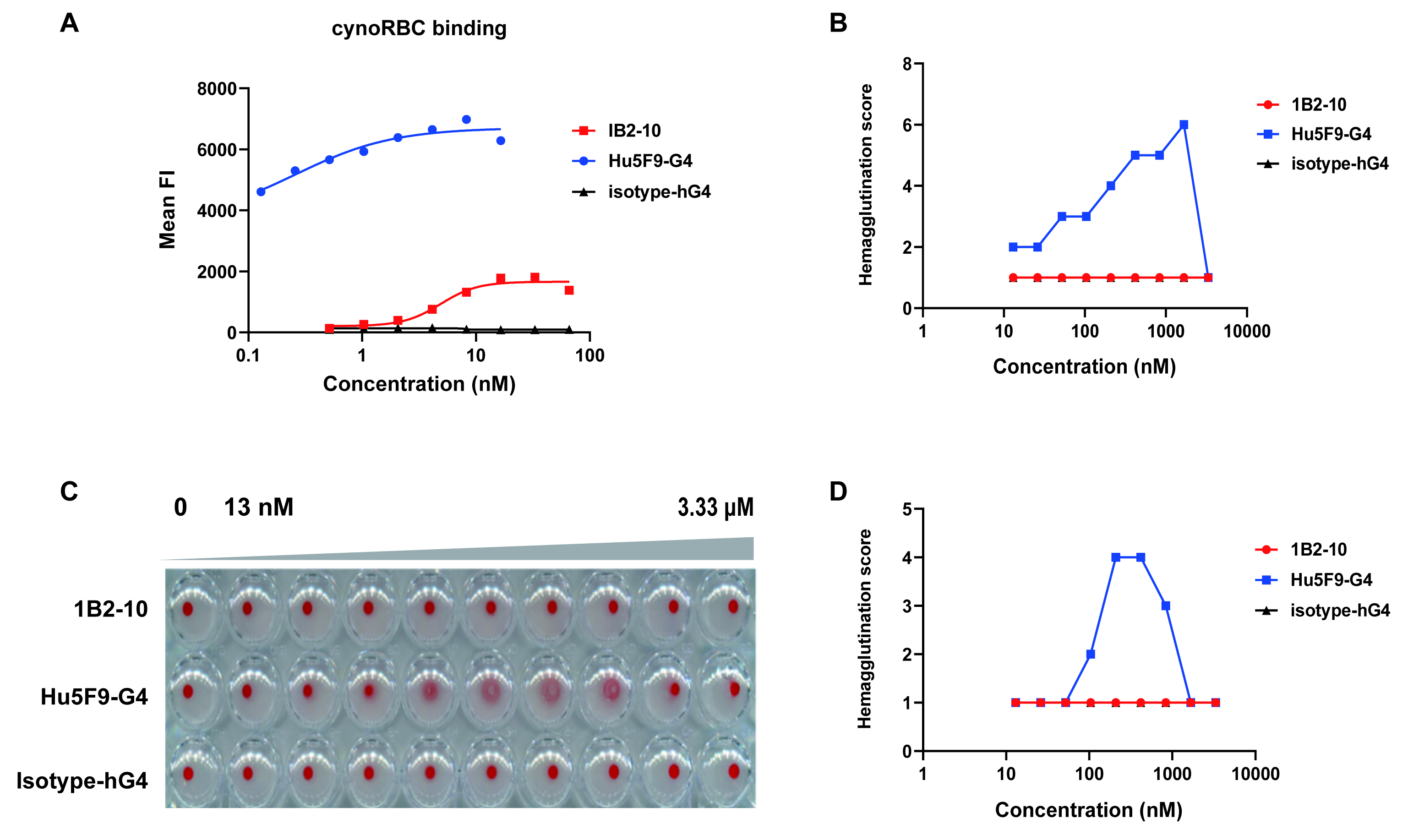


**Figure S2** 1B2-10 does not induce hemagglutination of cynomolgus monkey erythrocytes**. (A)** Flow cytometric analysis of 1B2-10 and Hu5F9-G4 binding to cynomolgus monkey red blood cells. (**B**) Human erythrocyte agglutination scores for 1B2-10 and Hu5F9-G4, based on observations from figure 2C. **(C)** Comparative analysis of hemagglutination effects: 1B2-10 demonstrates no hemagglutination of cynomolgus monkey erythrocytes, in contrast to Hu5F9-G4, which exhibits significant agglutination. **(D)** Cynomolgus monkey erythrocyte agglutination scores for 1B2-10 and Hu5F9-G4, based on observations from panel (C).

**
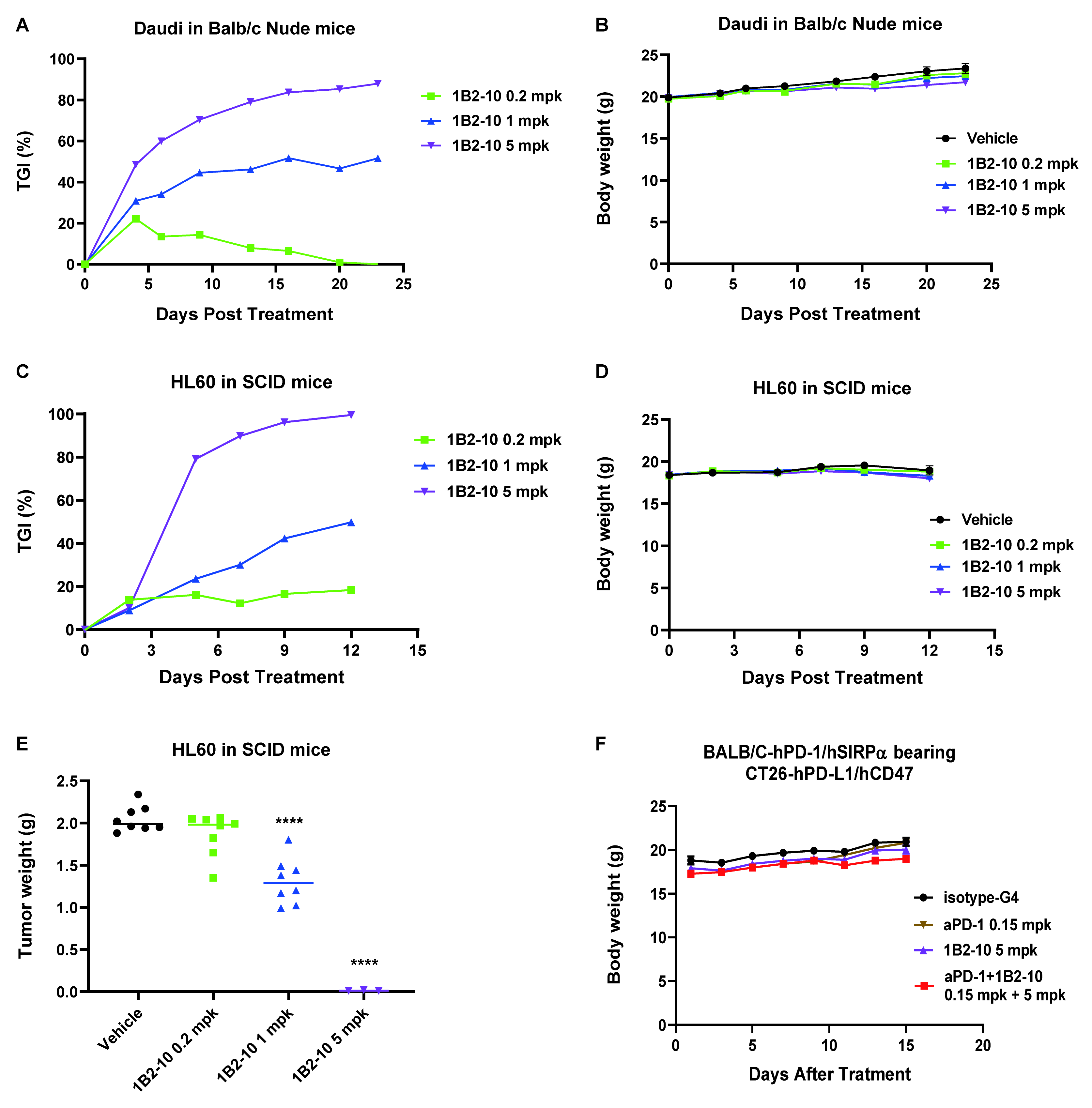
**

**Figure S3** 1B2-10 demonstrates potent tumor growth inhibition with favorable tolerability in multiple mouse models**. (A)** Tumor growth inhibition (TGI) and **(B)** body weight change in BALB/c nude mice subcutaneously inoculated with Daudi lymphoma cells. Mice were administered 1B2-10 at 0.2, 1.0, or 5.0 mg/kg, or vehicle control. **(C)** TGI, **(D)** body weight change, and **(E)** tumor weight change in SCID mice bearing HL-60 tumor cells. Mice received 1B2-10 at 0.2, 1.0, or 5.0 mg/kg, or vehicle control. Statistical comparisons between groups were performed using ordinary one-way ANOVA (****, *p* < 0.0001). **(F)** Body weight change in BALB/c-hPD-1/hSIRPα mice bearing CT26-hPD-L1/hCD47 tumors following treatment with indicated doses of 1B2-10 alone or in combination with anti-PD-1 antibody.


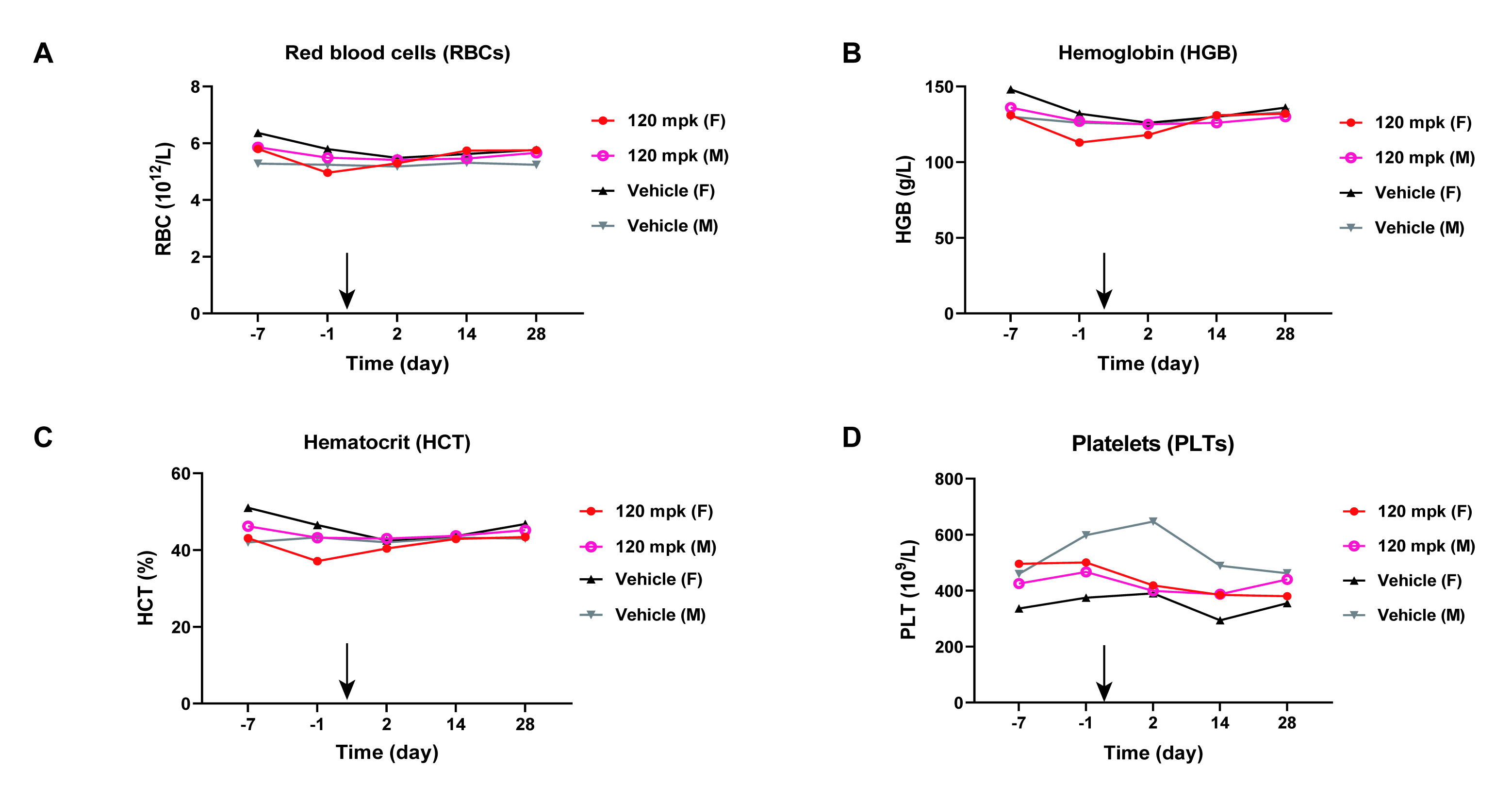


**Figure S4** Single-dose toxicology study of 1B2-10 in non-human primates. **(A, B, C, D)** Individual male (M) and female (F) cynomolgus monkeys were administered single intravenous infusions of 1B2-10 at a dose of 120 mg/kg. Red blood cell (RBC) count, hemoglobin (HGB) concentration, hematocrit (HCT), and platelets (PLTs) levels were monitored over a 4-week period.


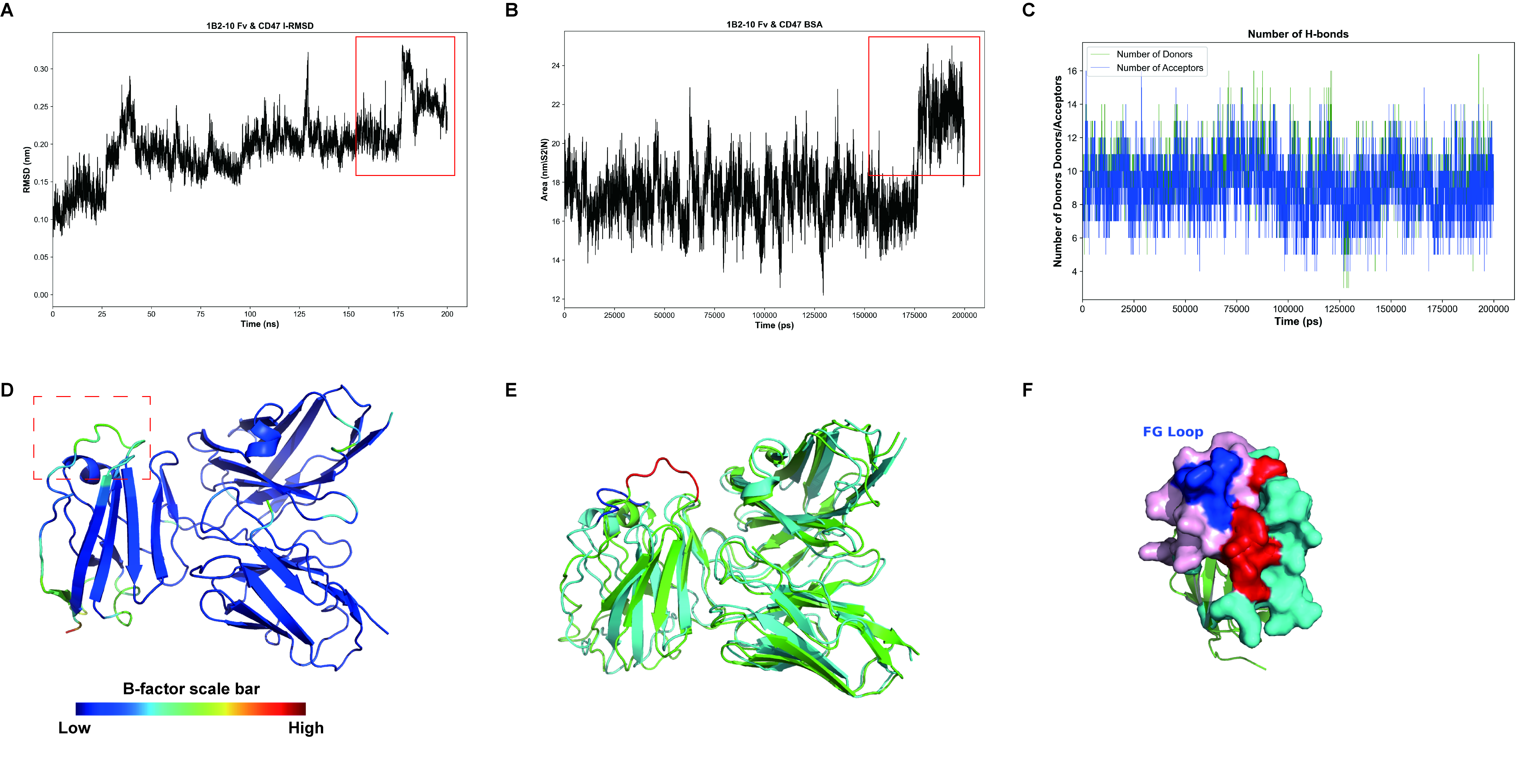


**Figure S5** The molecular dynamics simulation and epitope analysis of 1B2-10 Fv/CD47 complex. **(A)** Backbone RMSD of 1B2-10 Fv/CD47 complex over simulation time. **(B)** Buried surface area (BSA) of 1B2-10 Fv/CD47 complex throughout the simulation. **(C)** Number of hydrogen bonds (H-bonds) between donors (CD47) and acceptors (1B2-10 Fv) in the 1B2-10 Fv/CD47 complex over simulation time. The fluctuations in donor (CD47) and acceptor numbers are depicted in green and blue, respectively. **(D)** Atomic average B-factor distribution of the complex structure throughout the molecular dynamics simulation, represented by a color gradient from dark blue (lowest B-factor) to red (highest B-factor). The CD47-EAQNTT loop, highlighted in dark green and demarcated by a dashed red square, exhibits high structural flexibility. **(E)** Superposition of the complex structures at 150 ns (cyan) and 200 ns (green) of the MD simulation. The CD47-EAQNTT loop at 150 ns and 200 ns is highlighted in blue and red, respectively. **(F)** CD47 surface regions involved in binding to SIRPα and 1B2-10 Fv. Color coding: pink - regions binding only to SIRPα; cyan - regions binding only to 1B2-10 Fv; red - regions binding to both SIRPα and 1B2-10 Fv. The "FG loop" (blue) represents the blocking "hotspot".

Supplementary Table 1 Preclinical and Clinical landscape of CD47 antagonists

References

1. Liu J, Wang L, Zhao F, et al. Pre-Clinical Development of a Humanized Anti-CD47 Antibody with Anti-Cancer Therapeutic Potential. *PLoS One*. 2015;10(9):e0137345. doi:10.1371/journal.pone.0137345
2. Sikic BI, Lakhani N, Patnaik A, et al. First-in-Human, First-in-Class Phase I Trial of the Anti-CD47 Antibody Hu5F9-G4 in Patients With Advanced Cancers. J Clin Oncol. 2019;37(12):946-953. doi:10.1200/JCO.18.02018
3. Vyas P, Daver N, Chao M, et al. A Phase 2, Open-Label, Multiarm, Multicenter Study to Evaluate Magrolimab Combined with Antileukemia Therapies for First-Line, Relapsed/Refractory, or Maintenance Treatment of Acute Myeloid Leukemia. Blood. 2021;138(Supplement 1):3424. doi:10.1182/blood-2021-145584
4. Advani R, Flinn I, Popplewell L, et al. CD47 Blockade by Hu5F9-G4 and Rituximab in Non-Hodgkin’s Lymphoma. *N Engl J Med*. 2018;379(18):1711-1721. doi:10.1056/NEJM oa1807315
5. Evans TRJ, Italiano A, Eskens F, et al. Phase 1-2 study of TI-061 alone and in combination with other anti-cancer agents in patients with advanced malignancies. *JCO*. 2017;35(15_suppl):TPS3109-TPS3109. doi:10.1200/JCO.2017.35.15_suppl.TPS3109
6. Narla RK, Modi H, Bauer D, et al. Modulation of CD47-SIRPα innate immune checkpoint axis with Fc-function detuned anti-CD47 therapeutic antibody. *Cancer Immunol Immunother*. 2021;71(2):473-489. doi:10.1007/s00262-021-03010-6
7. Zeidan AM, DeAngelo DJ, Palmer J, et al. Phase 1 study of anti-CD47 monoclonal antibody CC-90002 in patients with relapsed/refractory acute myeloid leukemia and high-risk myelodysplastic syndrome. *Ann Hematol*. 2022;101(3):557-569. doi:10.1007/s00277-021-04734-2
8. Abrisqueta P, Sancho JM, Cordoba R, et al. Anti-CD47 Antibody, CC-90002, in Combination with Rituximab in Subjects with Relapsed and/or Refractory Non-Hodgkin Lymphoma (R/R NHL). *Blood*. 2019;134(Supplement_1):4089. doi:10.1182/blood-2019-125310
9. Peluso MO, Adam A, Armet CM, et al. The Fully human anti-CD47 antibody SRF231 exerts dual-mechanism antitumor activity via engagement of the activating receptor CD32a. *J Immunother Cancer*. 2020;8(1):e000413. doi:10.1136/jitc-2019-000413
10. Patnaik A, Spreafico A, Paterson AM, et al. Results of a first-in-human phase I study of SRF231, a fully human, high-affinity anti-CD47 antibody. *JCO*. 2020;38(15_suppl):3064-3064. doi:10.1200/JCO.2020.38.15_suppl.3064
11. Ni H, Cao L, Wu Z, et al. Combined strategies for effective cancer immunotherapy with a novel anti-CD47 monoclonal antibody. *Cancer Immunol Immunother*. 2021;71(2):353-363. doi:10.1007/s00262-021-02989-2
12. Liu D, Huang W, Chen B, Liu J. Anti-CD47 antibody and use thereof. 2023;(CN201810259545A).
13. Lakhani N, Orloff M, Fu S, et al. 295 First-in-human Phase I trial of IBI188, an anti-CD47 targeting monoclonal antibody, in patients with advanced solid tumors and lymphomas. J Immunother Cancer. 2020;8(Suppl 3). doi:10.1136/jitc-2020-SITC2020.0295
14. Garcia-Manero G, Erba HP, Sanikommu SR, et al. Evorpacept (ALX148), a CD47-Blocking Myeloid Checkpoint Inhibitor, in Combination with Azacitidine: A Phase 1 / 2 Study in Patients with Myelodysplastic Syndrome (ASPEN-02). *Blood*. 2021;138(Supplement 1):2601. doi:10.1182/blood-2021-146547
15. Kauder SE, Kuo TC, Harrabi O, et al. ALX148 blocks CD47 and enhances innate and adaptive antitumor immunity with a favorable safety profile. *PLOS ONE*. 2018;13(8):e0201832. doi:10.1371/journal.pone.0201832
16. Chow LQM, Gainor JF, Lakhani NJ, et al. A phase I study of ALX148, a CD47 blocker, in combination with standard anticancer antibodies and chemotherapy regimens in patients with advanced malignancy. JCO. 2020;38(15_suppl):3056-3056. doi:10.1200/JCO.2020.3 8.15_suppl.3056
17. Lakhani NJ, Chow LQM, Gainor JF, et al. Evorpacept alone and in combination with pembrolizumab or trastuzumab in patients with advanced solid tumors (ASPEN-01): a first-in-human, open-label, multicenter, phase 1 dose-escalation and dose-expansion study. *Lancet Oncol*. 2021;22(12):1740-1751. doi:10.1016/S1470-2045(21)00584-2
18. Lentz RW, Blatchford PJ, Hu J, et al. Results of a phase 2 study of evorpacept (E, ALX148), cetuximab (C), and pembrolizumab (P) in patients with refractory microsatellite stable metastatic colorectal cancer (MSS CRC). *JCO*. 2024;42(16_suppl):3530-3530.doi:10.120 0/JCO.2024.42.16_suppl.3530
19. Qu T, Zhong T, Pang X, et al. Ligufalimab, a novel anti-CD47 antibody with no hemagglutination demonstrates both monotherapy and combo antitumor activity. *J Immunother Cancer*. 2022;10(11):e005517. doi:10.1136/jitc-2022-005517
20. Miao M, Teng Q, Wu D, et al. AK117 (anti-CD47 monoclonal antibody) in Combination with Azacitidine for Newly Diagnosed Higher Risk Myelodysplastic Syndrome (HR-MDS): AK117-103 Phase 1b Results. *Blood*. 2023;142(Supplement 1):1865. doi:10.1182/blood-2023-179099
21. Gan HK, Coward J, Mislang ARA, et al. Safety of AK117, an anti-CD47 monoclonal antibody, in patients with advanced or metastatic solid tumors in a phase I study. *JCO*. 2021;39(15_suppl):2630-2630. doi:10.1200/JCO.2021.39.15_suppl.2630
22. Zeidan AM, Tong H, Xiao Z, et al. Trial in Progress: A Randomized, Double-Blind, Placebo-Controlled, Multicenter Phase 2 Study of AK117/Placebo in Combination with Azacitidine in Patients with Newly Diagnosed Higher-Risk Myelodysplastic Syndromes (AK117-205). Blood. 2024;144:6705. doi:10.1182/blood-2024-200541
23. Meng Z, Wang Z, Guo B, Cao W, Shen H. TJC4, a Differentiated Anti-CD47 Antibody with Novel Epitope and RBC Sparing Properties. *Blood*. 2019;134(Supplement_1):4063. doi:10.1182/blood-2019-122793
24. Berlin J, Harb W, Adjei A, et al. 385 A first-in-human study of lemzoparlimab, a differentiated anti-CD47 antibody, in subjects with relapsed/refractory malignancy: initial monotherapy results. *Journal for ImmunoTherapy of Cancer*. 2020;8:A410-A410. doi:10.1136/jitc-2020-SITC2020.0385
25. Xiao Z, Chang C, Li Q, et al. 617O Lemzoparlimab, a differentiated anti-CD47 monoclonal antibody, in combination with azacitidine (AZA) in patients with newly diagnosed higher risk myelodysplastic syndrome (HR-MDS): Initial clinical results. Annals of Oncology. 2022;33:S827. doi:10.1016/j.annonc.2022.07.743
26. Petrova PS, Viller NN, Wong M, et al. TTI-621 (SIRPαFc): A CD47-Blocking Innate Immune Checkpoint Inhibitor with Broad Antitumor Activity and Minimal Erythrocyte Binding. *Clinical Cancer Research*. 2017;23(4):1068-1079. doi:10.1158/1078-0432.CCR-16-1700
27. Ansell SM, Maris MB, Lesokhin AM, et al. Phase I Study of the CD47 Blocker TTI-621 in Patients with Relapsed or Refractory Hematologic Malignancies. Clinical Cancer Research. 2021;27(8):2190-2199. doi:10.1158/1078-0432.CCR-20-3706
28. Horwitz SM, Foran JM, Maris M, et al. Updates from Ongoing, First-in-Human Phase 1 Dose Escalation and Expansion Study of TTI-621, a Novel Biologic Targeting CD47, in Patients with Relapsed or Refractory Hematologic Malignancies. *Blood*. 2020;136:41-43. doi:10.1182/blood-2020-136198
29. Lin GHY, Viller NN, Chabonneau M, et al. Abstract 2709: TTI-622 (SIRPα-IgG4 Fc), a CD47-blocking innate immune checkpoint inhibitor, suppresses tumor growth and demonstrates enhanced efficacy in combination with antitumor antibodies in both hematologic and solid tumor models. *Cancer Research*. 2018;78(13_Supplement):2709-2709. doi:10.1158/1538-7445.AM2018-2709
30. Patel K, Maris MB, Cheson BD, et al. Ongoing, first-in-human, phase I dose escalation study of the investigational CD47-blocker TTI-622 in patients with advanced relapsed or refractory lymphoma. *JCO*. 2020;38(15_suppl):3030-3030.doi:10.1200/JCO.2020.38.15 _suppl.3030
31. Patel K, Ramchandren R, Maris MB, et al. Investigational CD47-Blocker TTI-622 Shows Single-Agent Activity in Patients with Advanced Relapsed or Refractory Lymphoma: Update from the Ongoing First-in-Human Dose Escalation Study. *Blood*. 2020;136:46-47.
32. Wang T, Wang SQ, Du YX, et al. Gentulizumab, a novel anti-CD47 antibody with potent antitumor activity and demonstrates a favorable safety profile. *Journal of Translational Medicine*. 2024;22(1):220. doi:10.1186/s12967-023-04710-6
33. Puro RJ, Bouchlaka MN, Hiebsch RR, et al. Development of AO-176, a Next-Generation Humanized Anti-CD47 Antibody with Novel Anticancer Properties and Negligible Red Blood Cell Binding. *Molecular Cancer Therapeutics*. 2020;19(3):835-846. doi:10.1158/1 535-7163.MCT-19-1079
34. Wilson WC, Bouchlaka MN, Capoccia BJ, et al. Therapeutic Potential of AO-176, a Next Generation Humanized CD47 Antibody, for Hematologic Malignancies. *Blood*. 2018;132:4180. doi:10.1182/blood-2018-99-118456
35. Burris III HA, Spira AI, Taylor MH, et al. A first-in-human study of AO-176, a highly differentiated anti-CD47 antibody, in patients with advanced solid tumors. *JCO*. 2021;39(15_suppl):2516-2516. doi:10.1200/JCO.2021.39.15_suppl.2516
36. Thaker YR, Rivera I, Pedros C, et al. A Novel Affinity Engineered Anti-CD47 Antibody With Improved Therapeutic Index That Preserves Erythrocytes and Normal Immune Cells. *Front Oncol*. 2022;12:884196. doi:10.3389/fonc.2022.884196
37. Yu J, Li S, Chen D, et al. SIRPα-Fc fusion protein IMM01 exhibits dual anti-tumor activities by targeting CD47/SIRPα signal pathway via blocking the “don’t eat me” signal and activating the “eat me” signal. *J Hematol Oncol*. 2022;15:167. doi:10.1186/s13045-022-01385-2
38. Qi J, Sun M, Ji D, et al. A First-in-Human Phase I Dose Escalation Study of IMM01, Sirpα Fc Protein in Patients with Relapsed or Refractory Lymphoma. Blood. 2022;140 (Supplement 1):3651-3652. doi:10.1182/blood-2022-158359
39. Yang W, Gao S, Yan X, et al. Latest results of a phase 2 study of IMM01 combined with azacitidine (AZA) as the first-line treatment in adults with higher risk myelodysplastic syndromes (MDS). JCO. 2024;42(16_suppl):6510-6510. doi: 10.1200/JCO.2024.42.16 _suppl.6510
